# Short-acquisition-time JPRESS and its application to paediatric brain tumours

**DOI:** 10.1007/s10334-018-0716-6

**Published:** 2018-11-20

**Authors:** Dominic Carlin, Ben Babourina-Brooks, Theodoros N. Arvanitis, Martin Wilson, Andrew C. Peet

**Affiliations:** 1 Institute of Cancer and Genomic Sciences, University of Birmingham, Birmingham, West Midlands UK; 2 Birmingham Women’s and Children’s Hospital NHS Foundation Trust, Birmingham, West Midlands UK; 3 Institute of Digital Healthcare, WMG, University of Warwick, Coventry, UK; 4 Centre for Human Brain Health, School of Psychology, University of Birmingham, Birmingham, West Midlands UK; 5 Clinical Research Block, Institute of Child Health, Whittall Street, Birmingham, B4 6NH UK

**Keywords:** Magnetic resonance spectroscopy, Metabolism, Brain neoplasms

## Abstract

**Objective:**

To develop and assess a short-duration JPRESS protocol for detection of overlapping metabolite biomarkers and its application to paediatric brain tumours at 3 Tesla.

**Materials and methods:**

The short-duration protocol (6 min) was optimised and compared for spectral quality to a high-resolution (38 min) JPRESS protocol in a phantom and five healthy volunteers. The 6-min JPRESS was acquired from four paediatric brain tumours and compared with short-TE PRESS.

**Results:**

Metabolite identification between the 6- and 38-min protocols was comparable in phantom and volunteer data. For metabolites with Cramer–Rao lower bounds > 50%, interpretation of JPRESS increased confidence in assignment of lactate, myo-Inositol and scyllo-Inositol. JPRESS also showed promise for the detection of glycine and taurine in paediatric brain tumours when compared to short-TE MRS.

**Conclusion:**

A 6-min JPRESS protocol is well tolerated in paediatric brain tumour patients. Visual inspection of a 6-min JPRESS spectrum enables identification of a range of metabolite biomarkers of clinical interest.

**Electronic supplementary material:**

The online version of this article (10.1007/s10334-018-0716-6) contains supplementary material, which is available to authorized users.

## Introduction

Metabolic reprogramming is an emerging hallmark of cancer [1]. Magnetic resonance spectroscopy (MRS) provides a convenient non-invasive means to investigate the metabolic composition of cancer. A wide range of metabolic changes have been identified in cohort studies of brain tumours [2, 3]; however, unambiguous detection of some overlapping J-coupled metabolites in a single spectrum has been limited. Clinical applications of MRS have, therefore, typically focused on N-acetyl aspartate (NAA), lipids, creatine (Cr) and choline (Cho), which can be identified at short and long TE, and lactate (Lac), which can be distinguished from lipids and macromolecules at long TE.

In addition to the above, there is growing evidence that glutamate (Glu) [3, 4], glutamine (Gln) [5], glycine (Gly) [3, 6, 7], myo-Inositol (mI) [3, 8–10], scyllo-Inositol (Scy) [4] and taurine (Tau) [2, 9–11] are altered in paediatric brain tumours. Therefore, these metabolites may provide new markers of diagnosis, prognosis and identification of high-risk patients, which may allow for better treatment stratification and more effective disease management. However, identification of these metabolites is hindered by small peak intensities and spectral overlap leading to reduced reproducibility [12] and increased Cramer–Rao lower bounds (CRLB) [13]. Various methods have been proposed for the detection of Glu, Gln, Gly, mI and Tau. These methods range in complexity from simply using an optimised TE [14–16] to more complex acquisition schemes such as TE averaging [17] and quantum filters [18–20]. However, these methods are typically optimised for the detection of a specific metabolite and can sacrifice information from other relevant metabolite biomarkers. As use of all quantified metabolite data can improve the accuracy of tumour classification [21], acquisition methods that identify as many metabolites as possible are, therefore, preferred.

Commonly used in vitro, J-resolved spectroscopy (JPRESS) [22, 23] has previously shown promise in brain tumours [24]. JPRESS is acquired by collecting PRESS spectra at multiple echo times, retaining the chemical shift information typical of conventional one-dimensional spectroscopy in F2, the *x*-dimension, and indirectly encoding the scalar coupling information for each metabolite in F1, the *y*-dimension. This spreads coupled metabolite resonances in two dimensions, disambiguating the identification of metabolites using the known chemical shift and scalar coupling constants for each metabolite based on their position in the spectrum [25].

One of the key challenges to implementing JPRESS in clinical practice is the associated long acquisition times. Children are often unable to tolerate long scanning sessions, making scan duration, particularly, pertinent in this setting. The aim of this study was to propose and test a short-duration JPRESS protocol, and evaluate its ability to detect and discriminate between overlapping metabolites of interest in paediatric brain tumours.

### Materials and methods

The study was approved by the East Midlands—Derby Research Ethics Committee (REC 04/MRE04/41) operating under the rules of Declaration of Helsinki 1975 (and as revised in 1983), and informed consent was obtained from all volunteers and patients. The acquisition protocol was optimised using a combination of simulated, phantom and volunteer data to determine the optimal spacing between TEs (ΔTE), number of TE steps acquired (NTE) and the number of averages (NSA) acquired per TE (NSA/TE). A high-resolution 38.4-min JPRESS protocol was used for comparison to assess the reduction in spectral quality (Table 1).

**Table 1:** Acquisition protocols in phantom and volunteer for JPRESS protocol and processing development

	TR (ms)	Starting TE (ms)	NSA/TE	TE spacing (ms)	#TEs	Maximum TE (ms)	Acquisition time (min)	Volunteers scanned
Protocol 1	2000	36	8	5	128	676	38.4	0 (2 Phantoms)
Protocol 2	2000	36	16	10	64	186	38.4	1
Protocol 3	2000	36	8	15	18	291	6	4 (2 Phantoms)

All data were acquired with one dummy scan per TE and a water-unsuppressed spectrum collected with NSA 1 per TE

### Experimental

#### Simulations

JPRESS spectra of common brain metabolites [25] were simulated using VESPA [26]. 1D MRS of each metabolite was simulated using the VESPA pulse sequence option ‘PRESS Ideal’ at a range of echo times from 35 ms to 675 ms. A fixed TE1 (20 ms) was used with TE2 varied as required for each simulated TE. The standard PRESS sequence was repeated for each TE and the 1D MRS was combined with JPRESS datasets with ΔTEs of 5, 10, 15 and 20 ms. The JPRESS spectra were then visually assessed to determine the optimal TE spacing. The PRESS Ideal pulse sequence assumes ideal hard RF pulses which flip all spins during an infinitely short time with the same phase and angle. Experimental acquisitions may be subject to signal cancellation due to phase variation and chemical shift displacement artefact for coupled spins such as lactate. In particular, the limited RF pulse bandwidth leads to spatially dependent evolution of J-coupling and consequently, additional artefact peaks at *J* = 0 Hz due to J-refocusing [27]. In general, however, the simulated JPRESS serves as a good approximation of metabolite peak patterns.

The 2D JPRESS spectra were further used as a reference library to aid metabolite identification and are presented in Online Resource 1; however, the spectrum for NAAG, which is typical of low abundance, does not include its associated coupled resonances. Each metabolite spectrum was displayed with 40 contour lines up to a level of 50% of the maximum signal intensity for that metabolite and no minimum threshold.

#### Phantom

MRS was acquired using a Philips Achieva 3T scanner (Philips Healthcare, Best, Netherlands). JPRESS was acquired as a TE series and TE1 was fixed to 20 ms for experimentally acquired data. All averages for a given TE were acquired before acquiring data for the next linear TE, with the Philips function to mitigate the frequency drift applied.

Phantom data were acquired from two phantoms. A “braino” phantom (GE Medical Systems, Milwaukee, WI, USA) of common brain metabolites at physiological levels containing 12.5 mM NAA, 12.5 mM Glu, 10 mM Cr, 7.5 mM, mI, 5 mM Lac and 3 mM Cho was scanned. The second phantom contained 5 mM Glu, 5 mM Gln, 5 mM Gly and 5 mM mI to assess the resolution of key overlapping metabolites under experimental conditions.

1 g of sodium azide was added to each phantom as a biocide to prevent the growth of bacterial organisms. The phantoms were pH adjusted to 7.2 and 0.5 mM and gadopentetate dimeglumine (Magnevist^®^, Bayer HealthCare Pharmaceuticals, Berlin, Germany) was added to each phantom as a relaxation agent to shorten relaxation times to the physiological range.

#### Volunteer

Data were obtained from five healthy adults (three male and two female) with a mean age of 25 ± 2 years for the volunteer data. MRS of parietal grey matter was acquired from cubic voxels of size 30 × 30 × 30 mm^3^. The acquisition protocols for the phantom and volunteer data are presented in Table 1. Protocol 2 was assumed to be the gold standard for metabolite detection due to its greater number of NSA/TE and the long echo train length. The spectral quality of the other protocols was assessed with reference to Protocol 2.

### Protocol development

#### TE spacing

The spectral width of F1 is set by 1/ΔTE. Phantom data acquired with ΔTE = 5 ms (Protocol 1) were spliced to generate additional subsets with ΔTE of 10 ms, 15 ms and 20 ms with the same NSA/TE and range of TEs.

#### NSA/TE

Volunteer data acquired with 8 NSA/TE (Protocol 3) and 16 NSA/TE (Protocol 2) were acquired. The two spectra were compared to determine if the major resonances of Glu, Gln, and mI could be identified with fewer NSA/TE.

#### Number of TEs

Phantom and volunteer data with final TEs of 290 ms (Protocol 3) and 665 ms (Protocols 1 and 2) were compared to assess the effect that truncating the number of TEs acquired had on spectral resolution.

### Clinical JPRESS of paediatric brain tumours

Four patients with paediatric brain tumours were investigated (Table 2). The optimised JPRESS protocol was acquired following conventional imaging.

**Table 2:** Patient details of paediatric brain tumour patients studied with JPRESS

Age (years)	Sex	Tumour type
9.8	Male	Medulloblastoma
8.2	Male	Pilocytic astrocytoma
6.5	Female	Optic pathway glioma
10.6	Male	Diffuse intrinsic pontine glioma (DIPG)

JPRESS was acquired from cubic voxels of size 30 × 30 × 30 mm^3^ for all the cases with TR = 2000 ms, TE_min_ = 42 ms, NTE = 18, ΔTE = 15 ms and NSA/TE = 8. The total acquisition time was 6 min. PRESS with an echo time of 35 ms, TR of 2000 ms and 128 NSA was also acquired from the cases with medulloblastoma and diffuse intrinsic pontine glioma.

### Data processing and analysis

The data were processed in two steps. First, the 1D MRS of each acquired TE was extracted and analysed with TARQUIN v4.3.8 using the 1H Brain basis set [28]. TARQUIN estimates of the water peak position, ref, in the water-suppressed spectra and CRLBs were recorded.

**Fig. 1:**
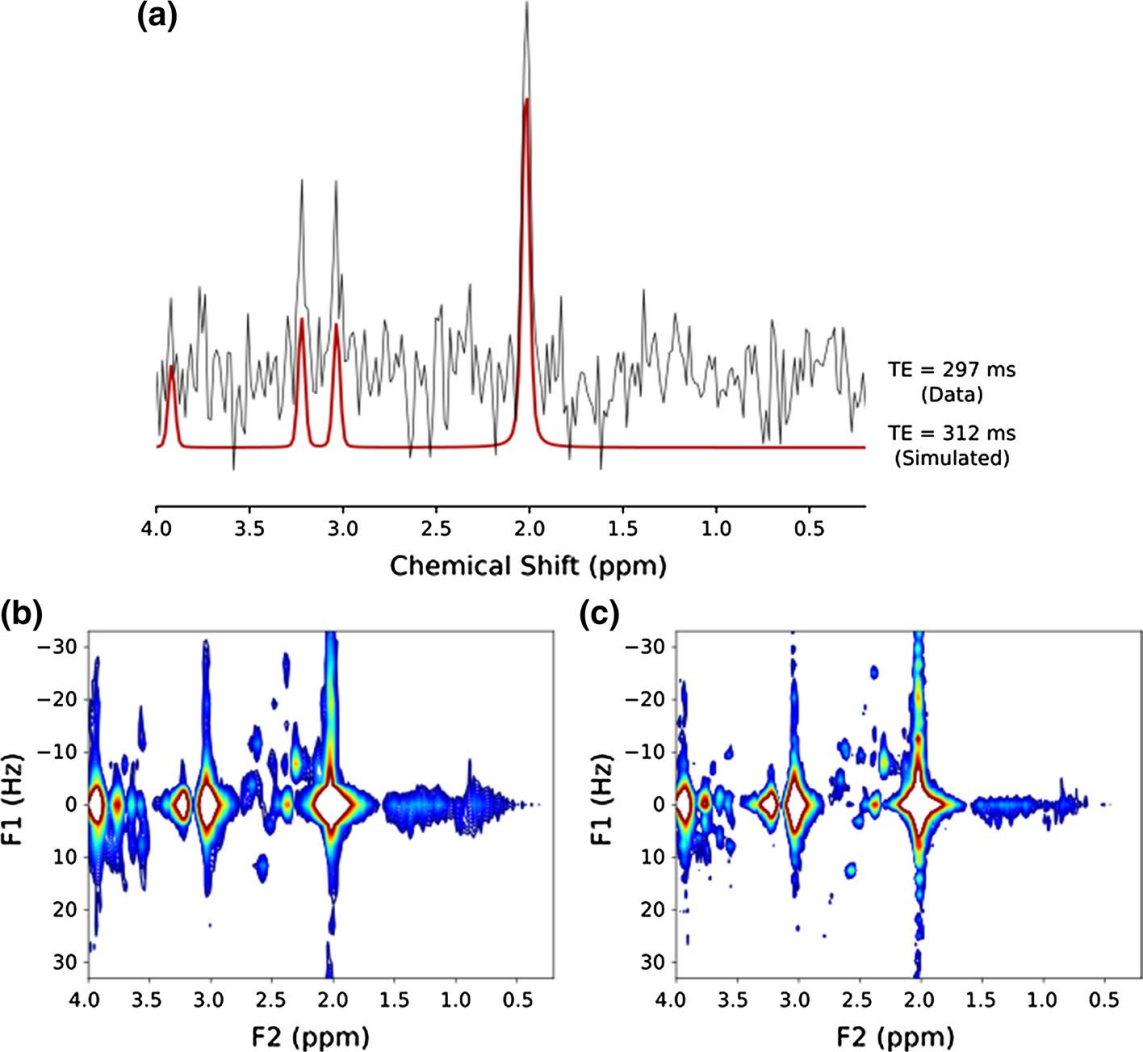
Representative examples of **a** acquired TE = 297-ms volunteer MRS from JPRESS acquisition and simulated TE = 312-ms MRS based on TARQUIN singlet fits to acquired TE = 297-ms MRS data. Simulated MRS was used to extend the JPRESS dataset. The F1 linewidths of **b** non-extended JPRESS were larger than those of **c** extended JPRESS for macromolecular resonances (0.5–1.5 ppm) and for coupled metabolites (2–3 ppm and 3.2–3.9 ppm)

The full multi-TE dataset for each experiment was then transferred to a personal computer and processed using software written in-house in Python (v 2.7.3) to produce the 2D JPRESS spectra. The water component of the free induction decays was removed using Hankel singular value decomposition and the baseline was corrected for any displacement to reduce interference. Baseline displacement correction was performed by taking the average value of a region of the spectrum containing only noise and no signal, and subsequently subtracting this value from the spectrum. Estimates of the water peak position, water_ref, in the water-suppressed spectra, were collected for each TE from the TARQUIN result file. Small frequency drifts between TEs caused broader resonances in the JPRESS F2 dimension. The water peak was, therefore, used as a chemical shift reference and was aligned to 4.65 ppm for all spectra in a dataset. Peak alignment was performed by multiplying each point, k, of the corresponding FID by exp(− 2*πi* × ref × *k*×(128⁄2000)), where ref = 4.65 ppm—water_ref, 128 MHz is the central 1H NMR frequency and 2000 Hz is the sampling frequency [29]. TARQUIN fits to NAA, Cho and Cr singlets which were used to simulate spectra at TE > 297 ms to reduce truncation artefacts caused by undersampling. Simulated spectra were appended to the dataset with a relaxation penalty of exp(− TE/T_2_) [30], applied to successive echo times and a T2 of 200 ms assumed for all metabolites [31]. Using this method, the dataset was extended from 18 TEs (297 ms) to 30 TEs (477 ms) improving spectral resolution (Fig. 1).

**Fig. 2:**
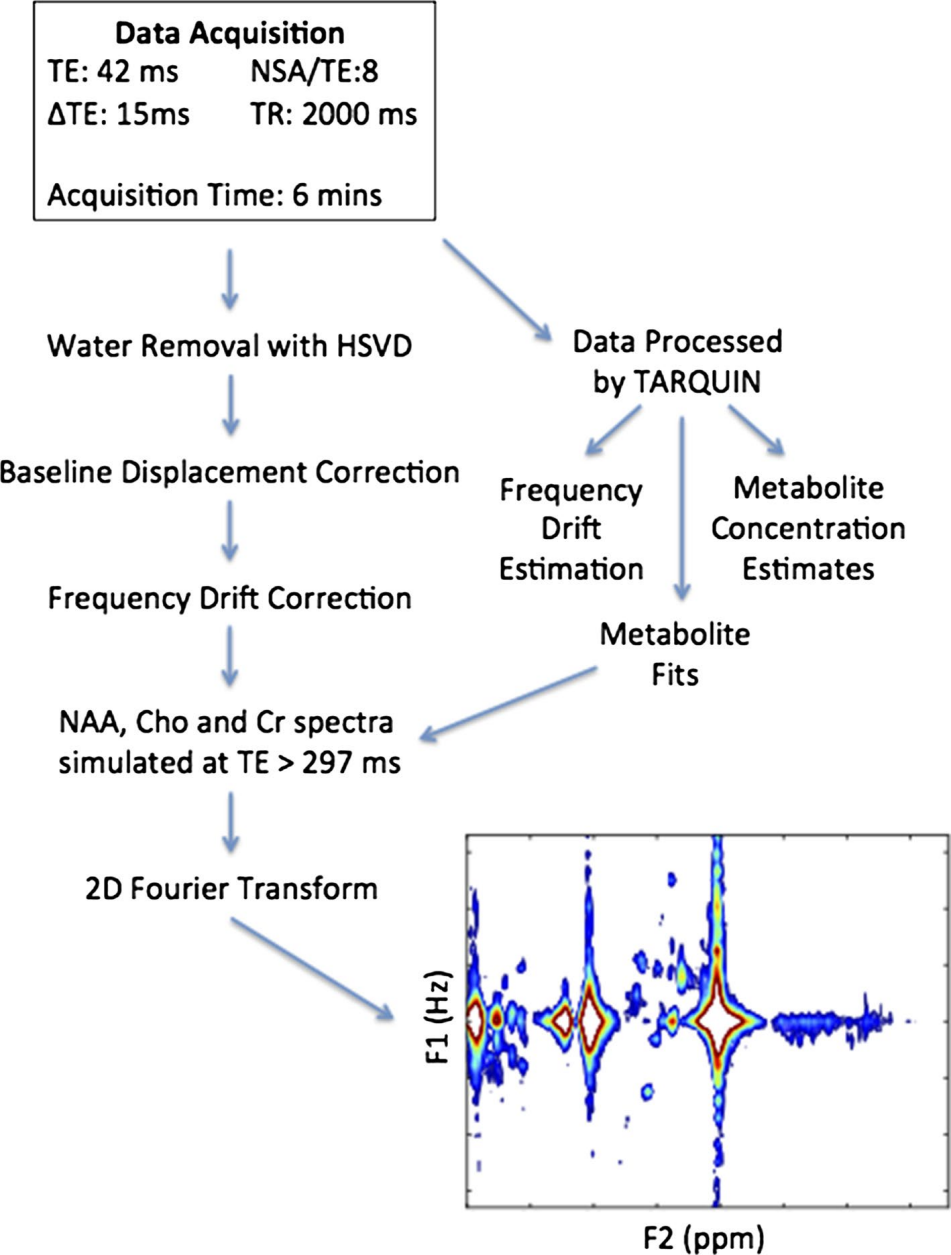
Optimised acquisition protocol and processing pipeline for JPRESS of paediatric brain tumours

The 2D Fourier-transformed JPRESS matrix was displayed as a contour plot in magnitude mode. Lower and upper bounds were used with thresholds adjusted to ensure spectra were clean and free of noise where possible. In each case, the thresholds were examined to determine if the metabolites could be identified in a more noisy spectrum. Spectral quality was determined through visual inspection of the display for artefacts. The thresholds of the display were further optimised manually to ensure that all desired peaks were present and fully resolved. The full data processing pipeline is shown in Fig. 2.

CRLBs were used to assess confidence in the presence of metabolites in short-TE PRESS.

## Results

### Protocol development and optimisation

The clinical JPRESS protocol was optimised for short acquisition times by optimising the parameter settings of ΔTE, NTE and NSA/TE. The ability to detect key metabolites using short-duration 6-min JPRESS was compared with a 38.4-min JPRESS protocol, with the 38.4-min protocol assumed to be the gold standard for metabolite detection in this study.

#### Phantom

**Fig. 3:**
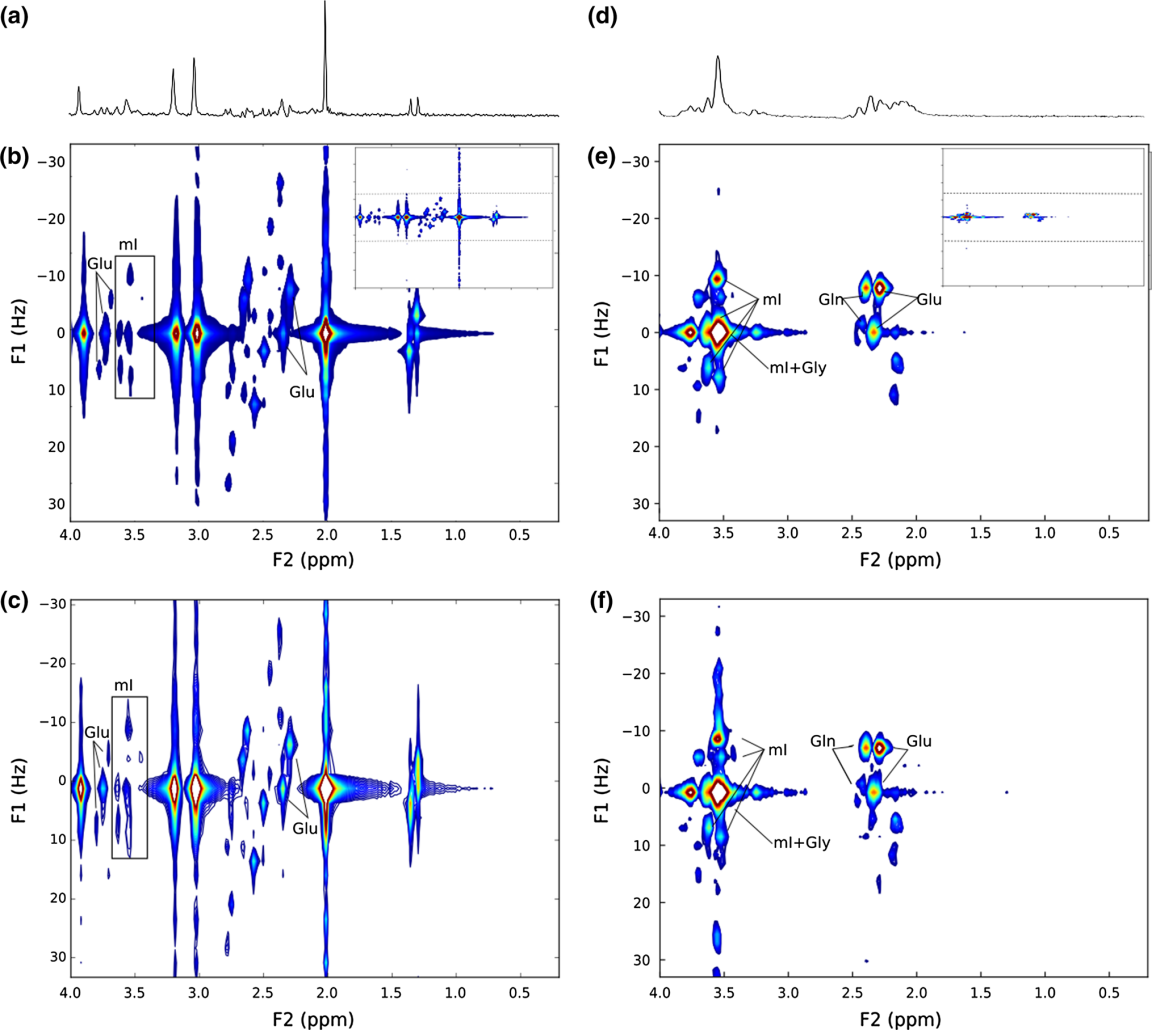
**a** 35-ms PRESS, **b** 38.4-min JPRESS (Protocol 1) and **c** 6-min JPRESS of the braino phantom (Protocol 3). **d** 35-ms PRESS, **e** 38.4-min JPRESS (Protocol 1) and **f** 6-min JPRESS (Protocol 3) of a phantom containing 5 mM Glu, 5 mM Gln, 5 mM Gly and 5 mM mI. For comparison of spectral quality, **b** and **e** show only the F1 spectral range from − 33 Hz to + 33 Hz, with the whole spectrum inset top right and dashed lines showing F1 = ± 33 Hz

Figure 3 shows short-TE MRS and JPRESS acquired in a braino phantom (Fig. 3a–c) and a phantom containing equal quantities of Glu, Gln, Gly and mI (Fig. 3d–f). The position of JPRESS resonance peaks can be described by their J-coupling (Hz) values and chemical shift (ppm) positions on the F1 and F2 axes, respectively. The Glu resonances with the greatest signal intensity are located at F2/F1 = 2.4 ppm/0 Hz and 2.35 ppm/− 7 Hz. mI presents as a complex arrangement of peaks between 3.5 and 3.6 ppm with characteristic resonances off the F1 = 0 Hz axis at F2/F1 = 3.50 ppm/− 10 Hz, 3.50 ppm/8 Hz and 3.60 ppm/5 Hz. The Gln resonance with the highest signal intensity is located at 2.40 ppm/− 7 Hz and Gly presents as a single resonance at 3.55 ppm/0 Hz.

A 38.4-min-long JPRESS protocol (Protocol 1) was compared with a shortened 6-min JPRESS protocol (Protocol 3). In both phantoms, all metabolite resonances fell within the F1 spectral range of ± 33 Hz and were visible using both protocols. Simulated JPRESS spectra of common brain metabolites are presented in Online Resource 1, and all metabolite resonances simulated also fall within this range, which corresponds to a ΔTE of 15 ms.

Improved resolution in the F1 dimension was found in the 38.4-min protocol (Fig. 3b) with the tails of the NAA, Cr and Cho singlets longer in the shorter 6-min protocol (Fig. 3c). There was no observable difference in resolution of the F2 dimension between the two protocols.

The main Glu and Gln resonances were resolved in both protocols. The Gly resonance at F2/F1 = 3.54 ppm/0 Hz was indistinct from mI; however, mI could be detected from its off − 0 Hz resonances.

### Volunteer

**Fig. 4:**
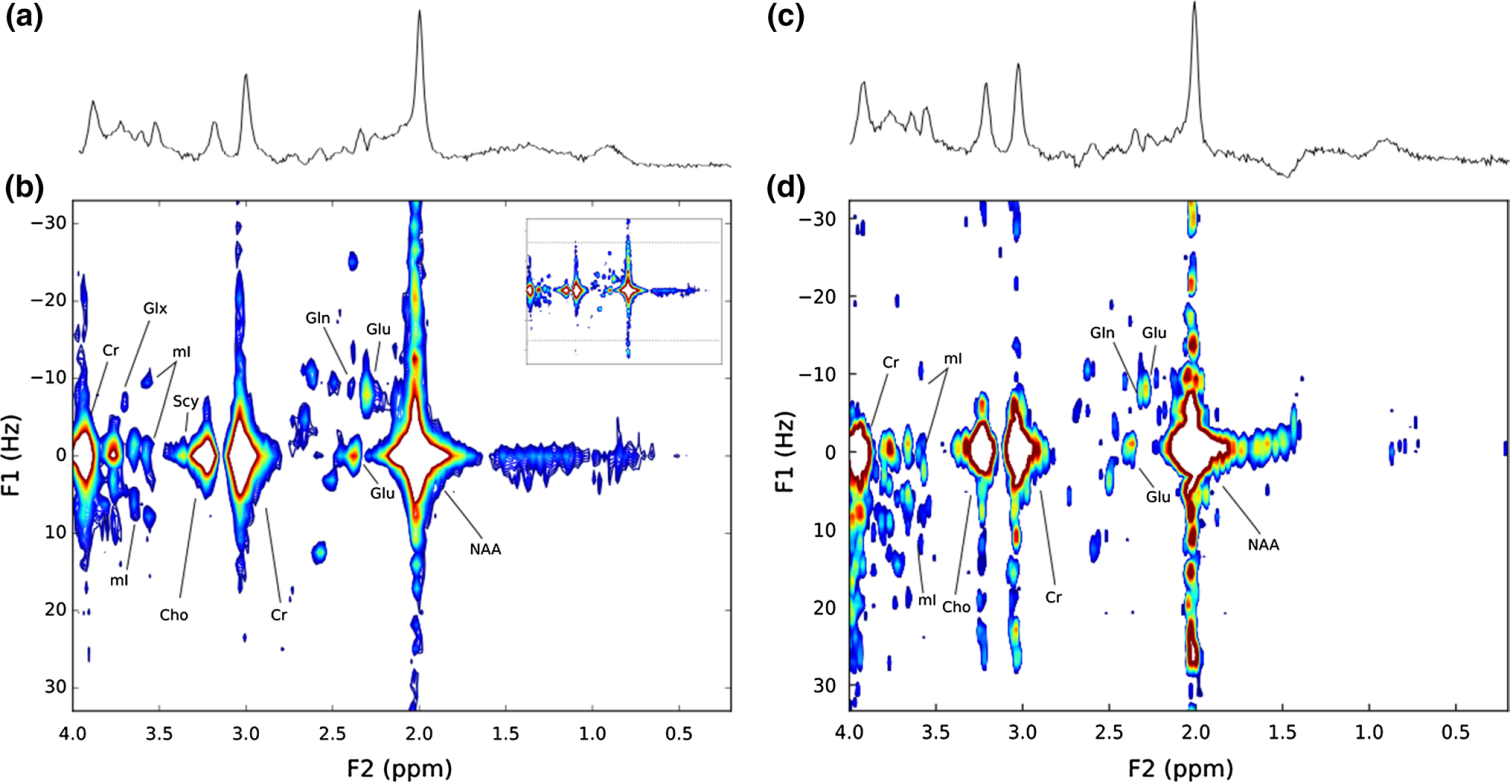
**a** 35-ms PRESS and **b** 38.4-min JPRESS (Protocol 2) of healthy grey matter in a female volunteer. **c** 35-ms PRESS and **d** 6-min JPRESS (Protocol 3) of a healthy grey matter in a male volunteer. For comparison of spectral quality, **b** shows only the F1 spectral range from − 33 Hz to + 33 Hz, with the whole spectrum inset top right and dashed lines showing F1 = ± 33 Hz

Figure 4 compares a 38.4-min JPRESS protocol (Fig. 4b) with a representative short 6-min protocol (Fig. 4d), and mI, Glu and Gln were identified by their characteristic resonance patterns in both the short and longer protocols; however, improved resolution of Glu and Gln was found in phantom data when compared to in vivo data. The 35-ms PRESS MRS (Fig. 4c) was unable to detect Gln, Tau or Gly; however, the corresponding short 6-min protocol discriminated Gln.

### JPRESS of paediatric brain tumours

**Fig. 5:**
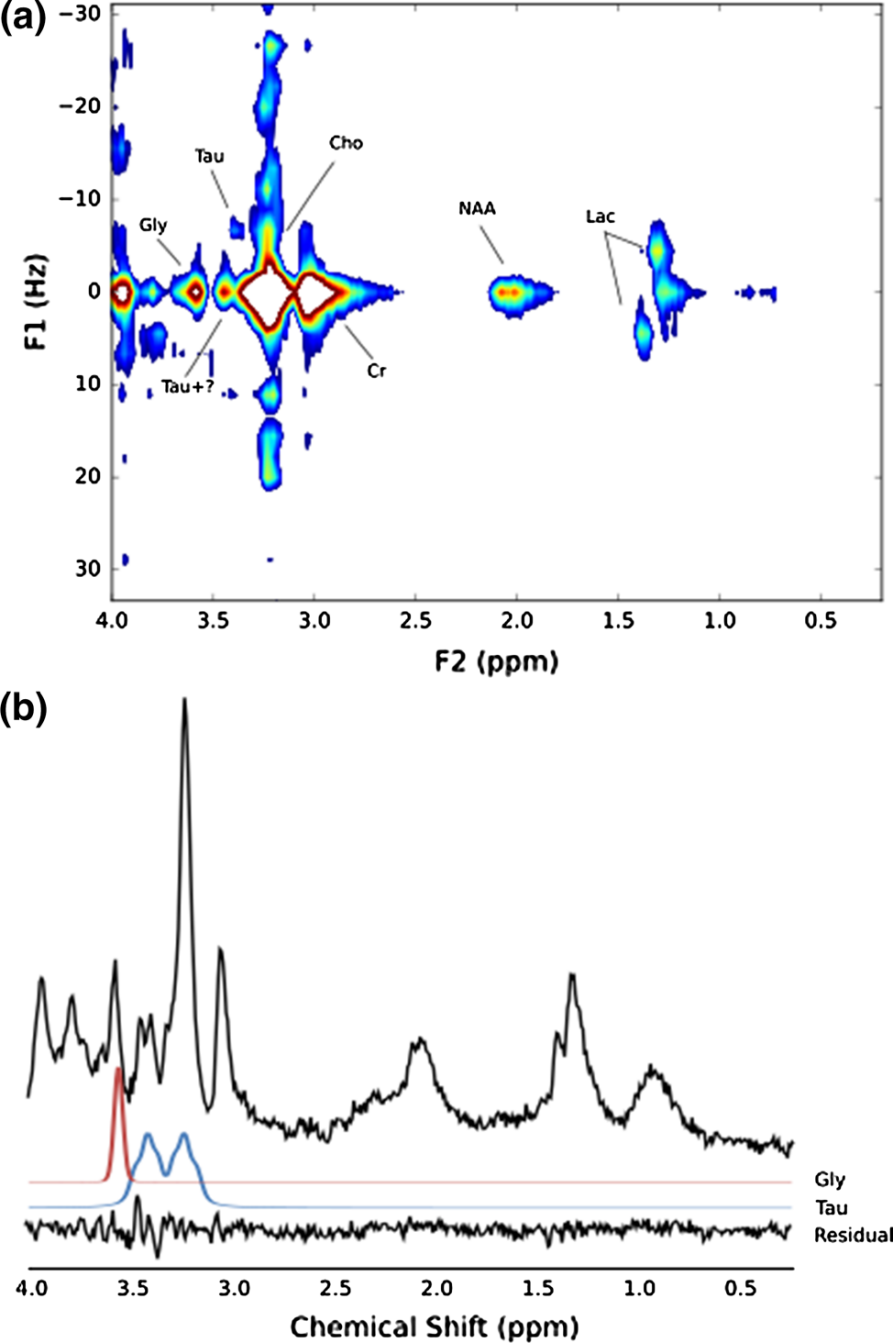
**a** JPRESS spectrum of medulloblastoma, **b** 35-ms PRESS of medulloblastoma collected in the same session. TARQUIN residual and fits (FWHM = 7 Hz) for Gly and Tau included

Figure 5 shows the JPRESS spectrum and a short-TE 35-ms PRESS spectrum acquired in the same scanning session from a medulloblastoma prior to treatment. The JPRESS resonance at F2/F1 = 3.54 ppm/0 Hz is most consistent with Gly, with no mI cross-peaks observed. The corresponding short-TE MRS resonance was assigned to Gly (CRLB = 12.9%) with no mI detected. Resonances were also present at F2/F1 = 3.40 ppm/0 Hz and 3.34 ppm/− 7 Hz. While the location of these resonances is most consistent with Tau, the F2/F1 = 3.40 ppm, 0 Hz resonance was twice as large as that at 3.34 ppm/− 7 Hz. The corresponding resonances of the simulated Tau JPRESS were of equal intensity. There is, therefore, an unassigned resonance at F2/F1 = 3.40 ppm, 0 Hz with no obvious associated resonances. The short-TE CRLB for Tau was 12.8%; however, there was a small residual peak in this region. The lactate doublet (short-TE CRLB = 112%) and lipid peak at 1.3 ppm (short-TE CRLB = 124%) were well separated in JPRESS. Glu (short-TE CRLB = 54.2%) and Gln (short-TE CRLB = 94.2%) were not detected in the JPRESS spectrum.

**Fig. 6:**
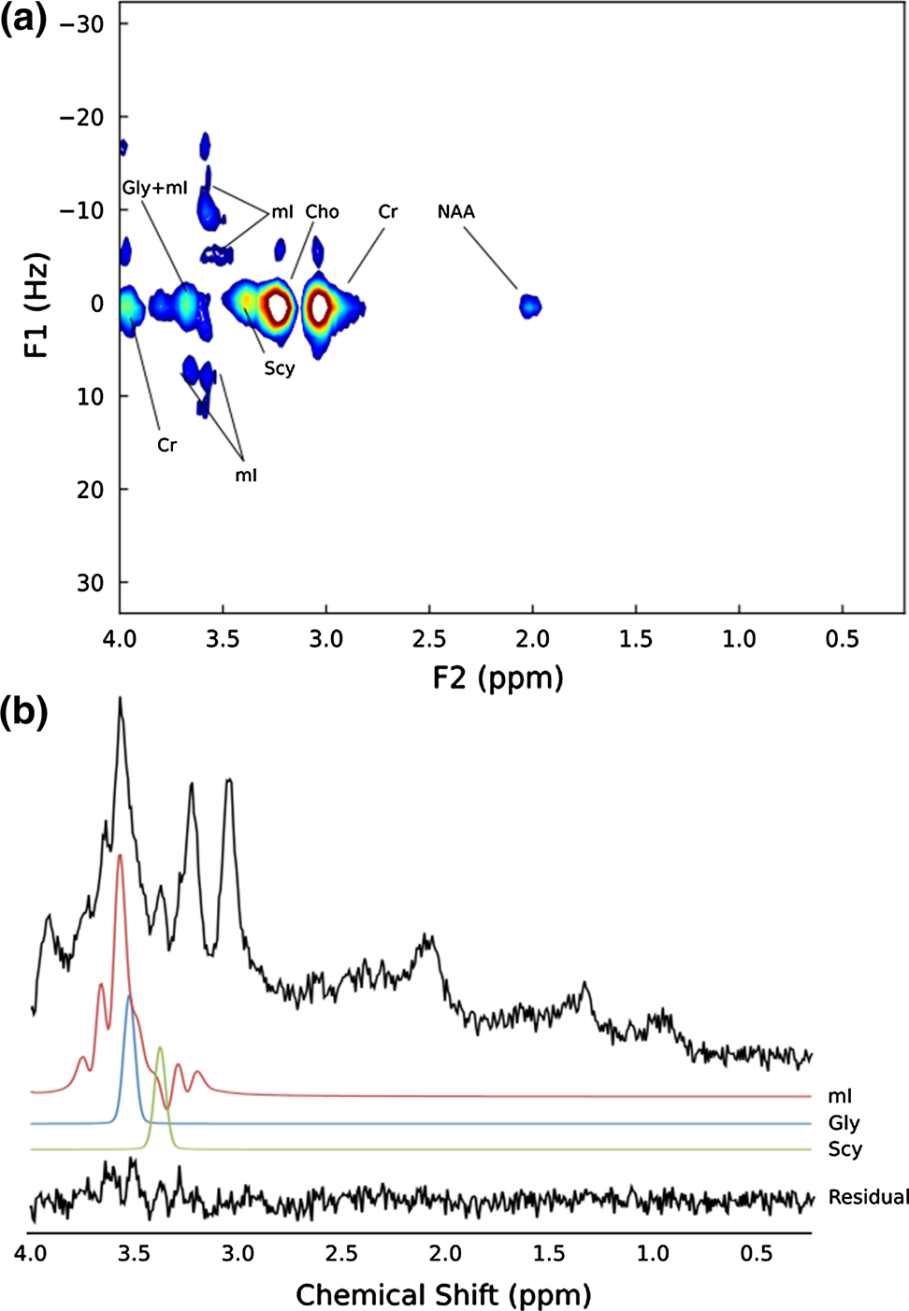
**a** JPRESS spectrum of pilocytic astrocytoma, **b** 1D MRS extracted from the pilocytic astrocytoma JPRESS dataset with a TE of 42-ms. TARQUIN fits (FWHM = 7 Hz) for mI, Gly and Scy included

Figure 6 shows the JPRESS spectrum and the extracted short-TE (42 ms) MRS of a pilocytic astrocytoma (SNR 12.8). The resonances between 3.5 and 3.6 ppm were assigned by TARQUIN to Gly (short-TE CRLB = 41.7%) and mI (short-TE CRLB = 53.5%) with the peaks at 3.55 ppm being of equal intensity. The intensities of the JPRESS resonances at F2/F1 = 3.54 ppm/0 Hz and 3.54 ppm/− 11 Hz are approximately equal, which is consistent with a combination of mI and Gly. Scy was also present in the JPRESS spectrum at 3.34 ppm/0 Hz and in the short-TE analysis (CRLB = 118%). Glu (CRLB = 67.1%) and Gln (not detected in short-TE MRS) were not detected in the JPRESS spectrum.

**Fig. 7:**
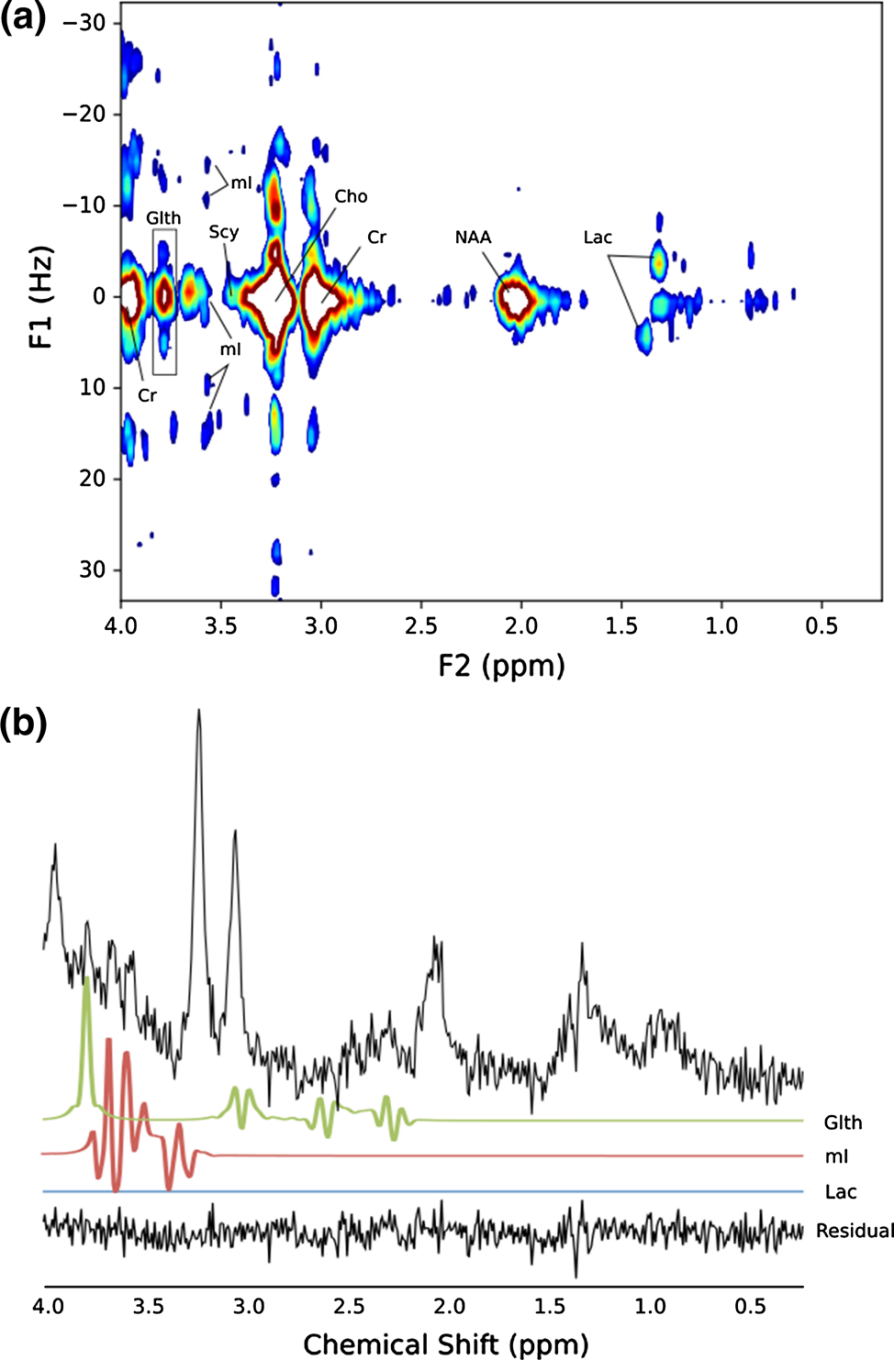
**a** JPRESS spectrum of optic pathway glioma (OPG), **b** 35-ms PRESS of OPG collected in the same session. TARQUIN residual and fits (FWHM = 4 Hz) for Lac, mI and Glth included

The JPRESS and corresponding short-TE (35 ms) MRS of an optic pathway glioma (OPG) are shown in Fig. 7. The short-TE assignments to the peaks at F1 = 0 Hz, F2 = 3.50, 3.6 and 3.8 ppm were to mI, mI and Glth with short-TE CRLBS of 67.3% for mI and 67.1% for Glth. The JPRESS shows the characteristic mI peak at F1/F2 = 3.50 ppm/− 10 Hz and also shows the splitting of the Lac doublet at 1.3 ppm. No Lac was detected in the short-TE MRS analysis. Glu (CRLB = 106%) and Gln (CRLB = 164%) were not detected in the JPRESS spectrum.

**Fig. 8:**
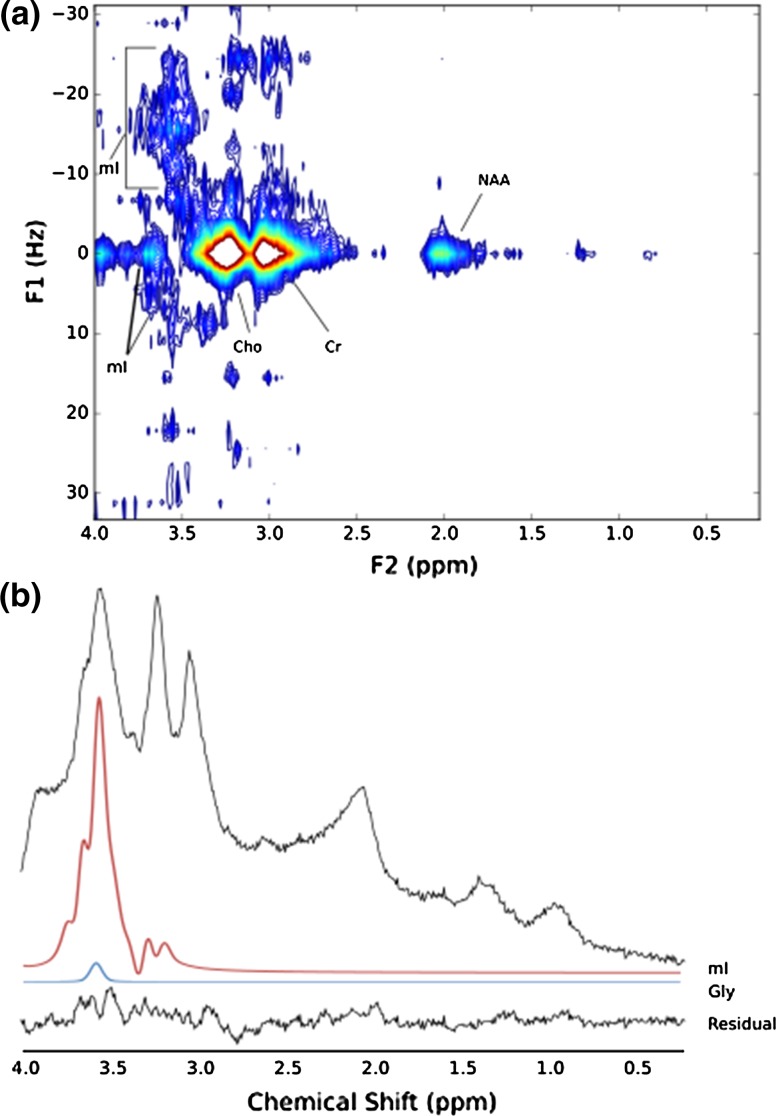
**a** JPRESS spectrum of diffuse intrinsic pontine gliomas (DIPG), **b** 35-ms PRESS of DIPG collected in the same session. TARQUIN residual and fits (FWHM = 8 Hz) for mI and Gly included

The JPRESS and short-TE PRESS spectra (SNR = 12.2) of a DIPG post-radiotherapy are shown in Fig. 8. The resonances between − 20 Hz and 10 Hz at approximately 3.54 ppm are most consistent with mI. The vast majority of this region in the short-TE PRESS was assigned to mI (CRLB = 13.2%) with a small amount of Gly (CRLB = 112%) present. Glu (CRLB = 134%) and Gln (CRLB = 110%) were not detected in the JPRESS spectrum. Since mI is a complex multiplet, a large FWHM leads to individual peaks merging and, therefore, a different overall lineshape being formed when compared with the cases where the FWHM is small and the individual peaks are better resolved. This effect is seen in the DIPG spectrum where the FWHM is 8 Hz (Fig. 8) compared to the OPG spectrum where the FWHM is 4 Hz (Fig. 7).

## Discussion

This study presents a 6-min clinical protocol for JPRESS developed for visual detection of key metabolites found in paediatric brain tumours and its application to a series of cases. The protocol was optimised using a braino phantom and volunteer data with the short-duration protocol’s efficacy assessed by comparing with a longer protocol which was taken as the reference standard.

The main metabolites of interest in this study were Glu, Gln, Gly, mI, Scy and Tau due to their roles in cancer metabolism. Metabolite identification was assessed in phantom and volunteer JPRESS spectra acquired with 8 NSA/TE and 16 NSA/TE. Glu, Gln and mI could all be identified in the 8 NSA/TE volunteer spectra and these were selected to reduce the scan duration. The absence of Gly, Scy and Tau was expected in volunteers due to their relative low abundance in healthy brain [25] but they are of sufficiently high concentration in some brain tumours that the same NSA/TE should be appropriate for these metabolites [2]. Further reduction of the protocol duration was obtained by considering the final TE acquired. Due to their relatively small peak intensities, a final TE of 297 ms was sufficient for strongly coupled resonances to be indistinguishable from the noise following T2 relaxation decay, but for singlets, approximately 20% of the metabolite signal remains assuming typical T2 relaxation times [29].

Not acquiring MRS with a TE greater than 297 ms reduced acquisition time and did not cause major artefacts in the JPRESS spectral when the singlet decay at longer echo times was simulated. The ΔTE increment was increased to 15 ms for a J-resolved bandwidth of 67 Hz, which was considered to be the bandwidth required to observe all coupled resonances of common metabolites [25]. While the ∆TE increment used here is sufficient for metabolite detection, smaller ∆TE increments are also used for JPRESS applications [22] as these can help avoid artefacts caused by signal folding due to insufficient water suppression or no or non-optimal apodisation.

In the four paediatric brain tumour cases, metabolites of interest were confirmed or excluded based on the JPRESS spectrum compared with the short-TE MRS. In the medulloblastoma case, Gly and Tau were identified with peak positions and J-coupled resonances consistent with metabolite simulations. The short-TE analysis was consistent with the interpretation of the JPRESS spectrum, with a small residual peak between 3.30 and 3.40 ppm. Based on the JPRESS appearance, this could be due to an unassigned singlet peak. The medulloblastoma JPRESS was consistent with ex vivo HR-MAS analysis [32]. Interpretation of JPRESS, with the aid of simulated data, also corroborated the TARQUIN assignments of Gly, mI and Scy in the short-TE MRS pilocytic astrocytoma patient case. TARQUIN analysis of the short-TE DIPG MRS indicated that mI was the predominant metabolite at 3.50 ppm, which was consistent with the JPRESS spectrum. In the optic pathway glioma patient, the JPRESS indicated that Glth, mI and Lac were present in the voxel, whilst TARQUIN’s assignments in the short-TE PRESS had CRLB > 50% for Glth and mI and Lac was not fitted. The discrimination of these metabolites of interest has clinical relevance for both diagnosis and prognosis. DIPG and OPG patients rarely have biopsies and, therefore, imaging plays a vital role in their tumour diagnosis. The discrimination of Gly and mI is of particular importance as Gly has been found to be a survival marker in paediatric brain tumours [33], while Scy is also a marker of survival [5]. The confidence in the assignment of these metabolites is, therefore, of value and JPRESS can be used in a short scan time to achieve this, with the features seen in the low-grade optic pathway glioma and pilocytic astrocytomas reflecting those commonly identified ex vivo [34].

One of the key advantages of JPRESS over short-TE MRS is that visual inspection increases confidence in metabolite assignment. CRLBs are the standard method of assessing confidence in 1D MRS metabolite assignments. While for reliable metabolite quantification, the consensus CRLB threshold is 20%, there is consensus that metabolites can be detected with high confidence when CRLBs are less than 50% [35]. However, in the short-TE analysis of patients in this study, several key metabolites of clinical interest had CRLBs > 50% but were clearly present in the JPRESS spectrum. These included Lac in the medulloblastoma and OPG, mI in the PA and OPG and Scy in the PA, with Gly also having a relatively high CRLB of 41.7% in the PA.

A range of methods are available for the detection of metabolite biomarkers of disease and prognosis, including optimised TE 1D MRS and other spectral-editing approaches. JPRESS was chosen ahead of these as 2D methods do not suppress or enhance metabolite resonances, allowing a wide range of metabolites to be potentially identified. JPRESS was also chosen ahead of L-COSY [36] as it is more readily available on clinical 3 T MR systems without modification and has regulatory approval (CE-marking) on the scanner used in this study; however, the analysis itself is not CE-marked. JPRESS also offers the facility to extract the short- and long-TE 1D MRS spectra that radiologists are already familiar with interpreting, aiding its translation into the clinical environment; however, these extracted spectra will have lower SNR than a typical 1D MRS acquisition. Further improvement of JPRESS could be conferred by optimising the apodisation function or by the use of a weighted averaging acquisition [37].

While the presented JPRESS protocol offers some clear advantages over conventional short-TE PRESS, there are a number of limitations to this study. First, with visual detection of key metabolite biomarkers being the aim of this study, no attempt was made to quantify metabolite concentrations. However, while quantification was not performed here, a recent study of short-duration JPRESS observed a test–retest reliability < 10% for NAA, Cho, Glth, Glu and mI [38]. 2D JPRESS can also be quantified using the designated quantification package ProFit [39], which could yield the same, or better results, as those achieved here by visual inspection. While ProFit was not used in this study, a comparison of the two approaches would be a logical extension to this work and will be examined in future work.

Second, a voxel of size 30 × 30 × 30 mm^3^ was used to ensure sufficient SNR. Though this voxel is large, this JPRESS protocol would be suitable for brain tumours which have not been resected, with metabolite-specific pulse sequences used to follow-up response to treatment for pre-selected metabolites. Recent advances, such as the combination of JPRESS with fast echo-planar spectroscopic imaging approaches [40, 41], are likely to mitigate the partial volume problems associated with large voxels. These methods typically require the use of a research mode on clinical scanners, but should also be investigated.

Third, while both Glu and Gln could be identified in healthy volunteers, they were not detected in any of the four paediatric brain tumours investigated with JPRESS. This is likely due to the lower abundance of these metabolites in brain tumours compared with age-matched normal brain [3]. In the corresponding short-TE MRS, Glu and Gln consistently had CRLBs > 50%; however, a greater NSA/TE might be better suited for the detection of these metabolites. While we did not thoroughly explore the effect of varying the NSA/TE, we chose to acquire eight averages per TE to maximise SNR. Reducing the NSA/TE may be able to achieve similar spectral quality in a shorter timeframe, while scanning more TEs with a smaller NSA/TE could potentially be used to improve spectral quality.

Finally, while JPRESS has the potential to identify or confirm the presence of novel metabolite biomarkers, metabolite assignment can still be a non-trivial task. It is hoped that the simulated JPRESS spectra presented in Online Resource 1 will aid others in basic metabolite assignment. High-resolution experiments using ex vivo tissue could help to elucidate metabolite spectra further.

## Conclusions

A 6-min JPRESS protocol was well tolerated in a paediatric setting and was able to detect metabolite biomarkers of disease and prognosis in childhood brain tumours. Visual inspection of JPRESS confirmed the presence of metabolites with high CRLBs in short-TE MRS and would be suitable for use in patients with large tumours.

## Electronic supplementary material

Below is the link to the electronic supplementary material.

Supplementary material 1 (PDF 738 kb)
