# Supplementary material for: Short-acquisition-time JPRESS and its application to paediatric brain tumours
Source: MAGMA. 2018 Nov 20;32(2):247–58. doi: 10.1007/s10334-018-0716-6 (PMC6424926; doi:10.1007/s10334-018-0716-6)
Supplement: Supplementary file 1 — Supplementary material 1 (PDF 738 kb) [file 10334_2018_716_MOESM1_ESM.pdf]

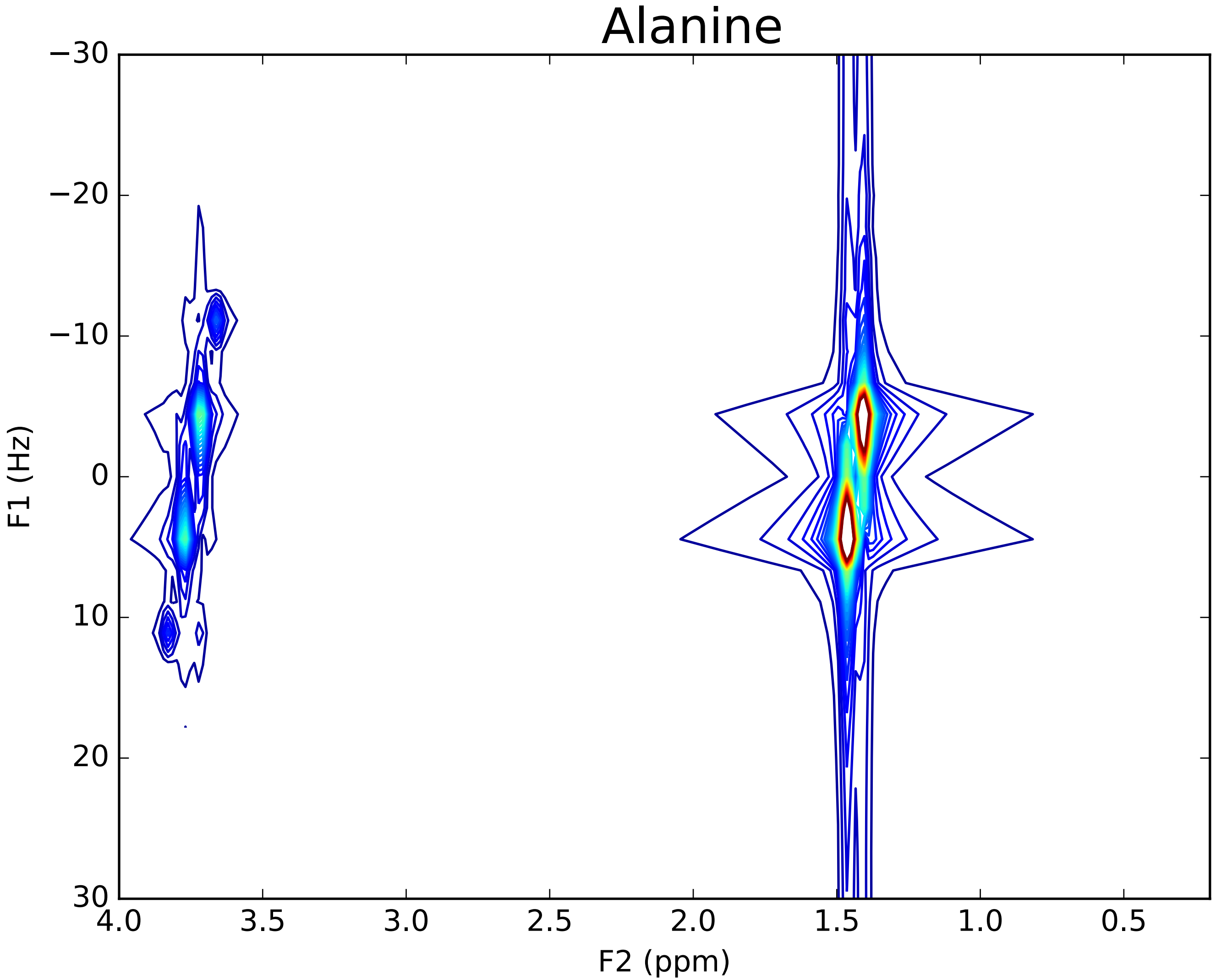
Alanine
F1 (Hz)
F2 (ppm)
−30
−20
−10
0
10
20
30
4.0
3.5
3.0
2.5
2.0
1.5
1.0
0.5

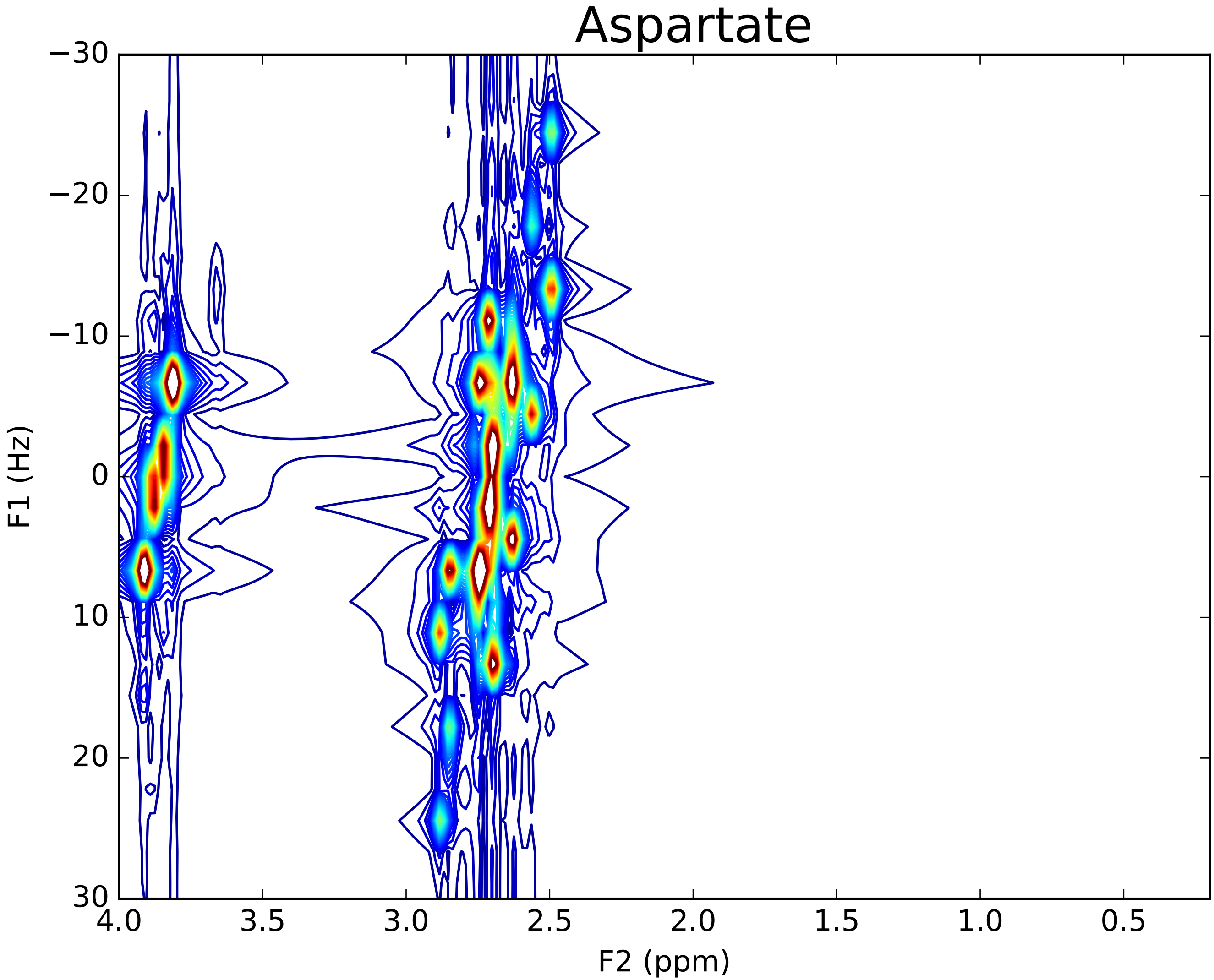
Aspartate
F1 (Hz)
F2 (ppm)
−30
−20
−10
0
10
20
30
4.0
3.5
3.0
2.5
2.0
1.5
1.0
0.5

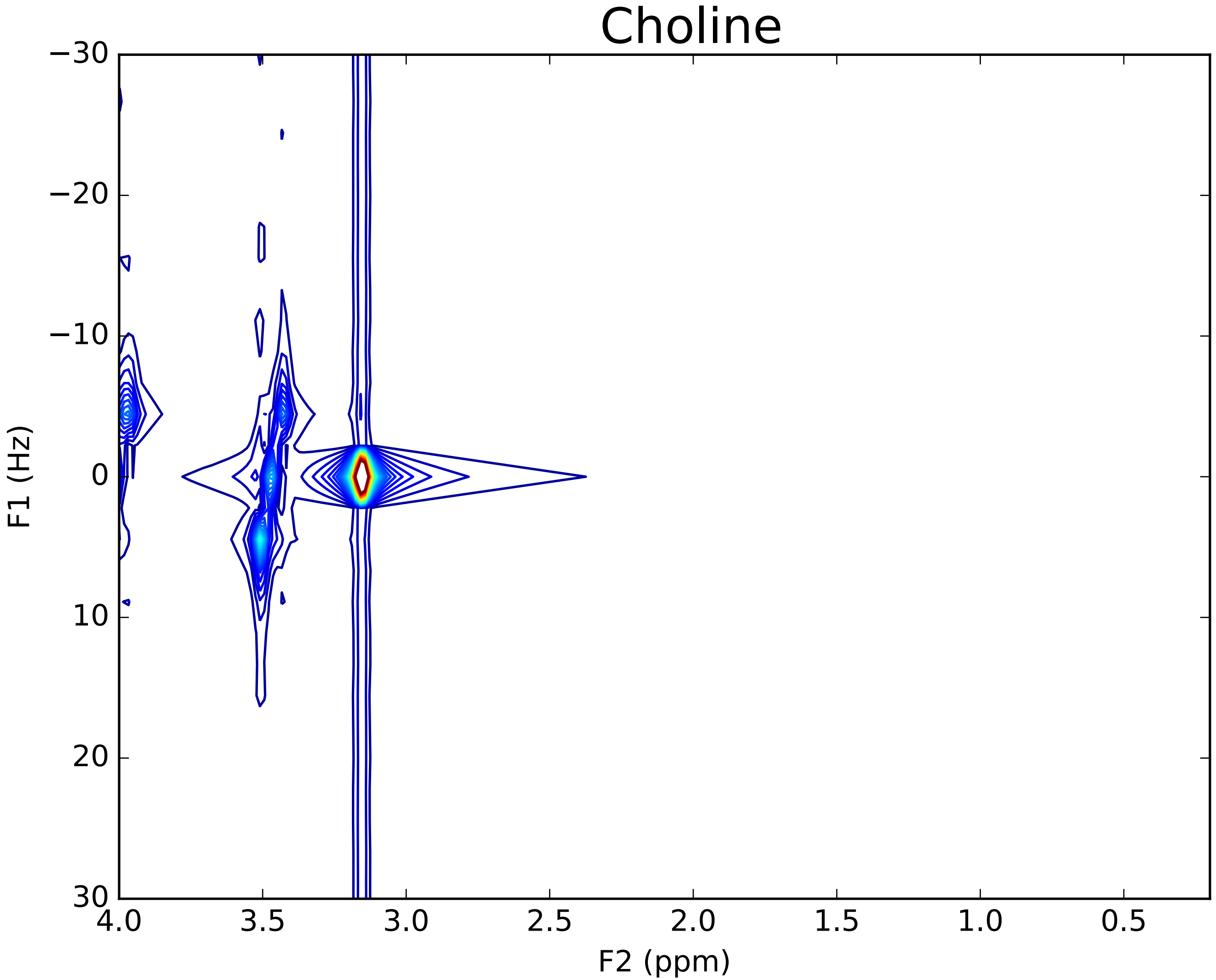
Choline
−30
−20
−10
0
10
20
30
F1 (Hz)
4.0
3.5
3.0
2.5
2.0
1.5
1.0
0.5
F2 (ppm)

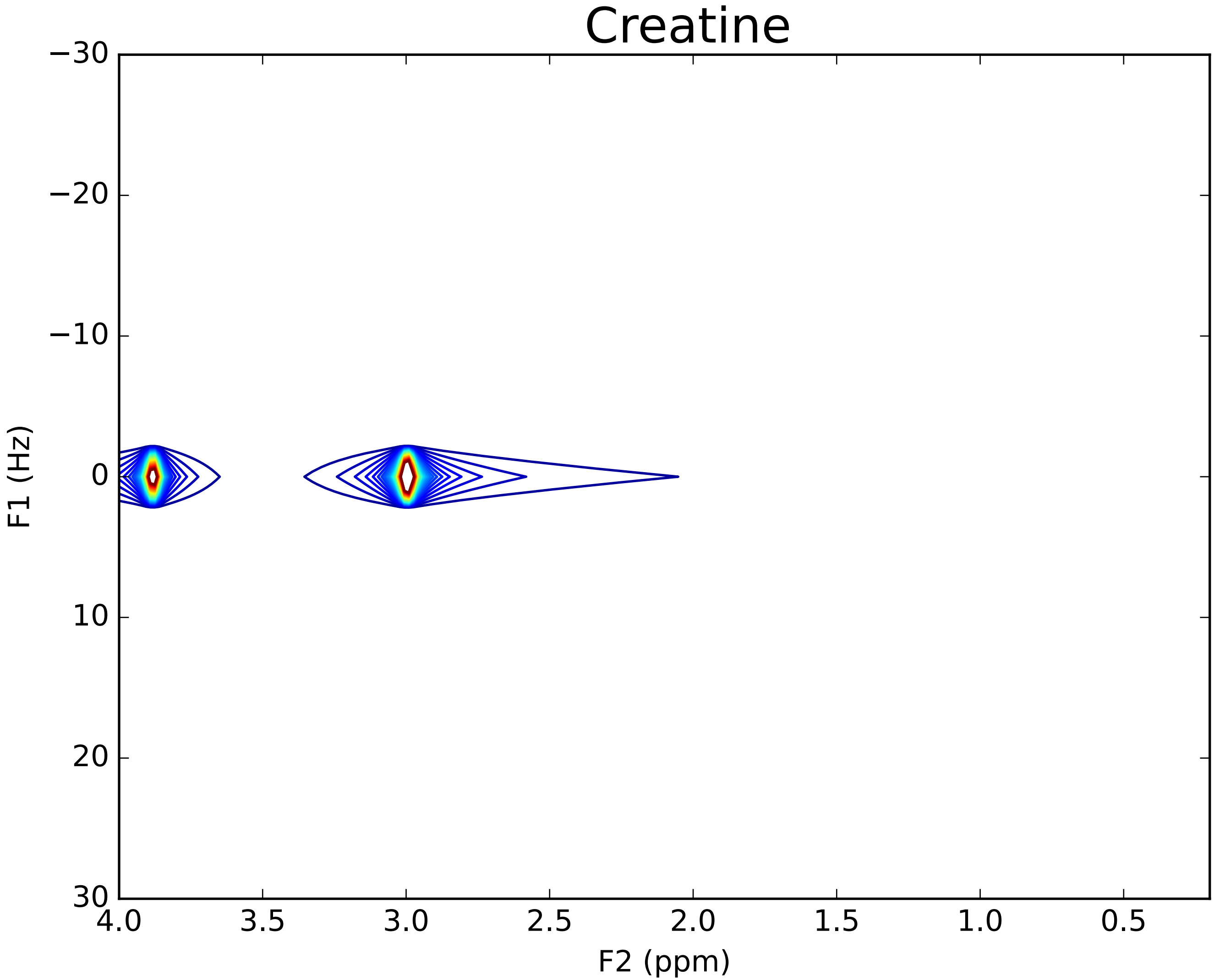

Creatine
−30
−20
−10
0
10
20
30
F1 (Hz)
4.0
3.5
3.0
2.5
2.0
1.5
1.0
0.5
F2 (ppm)

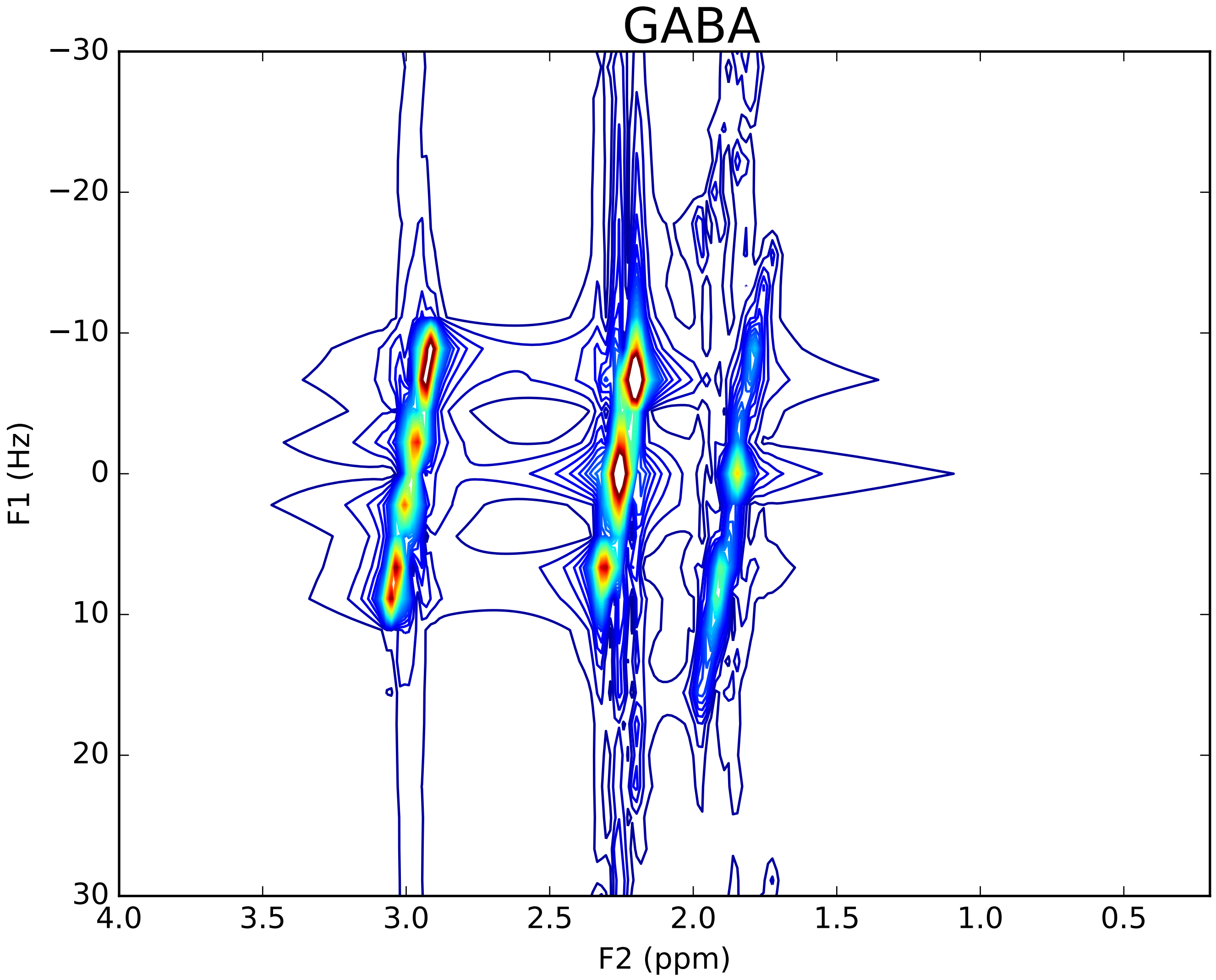

GABA
F1 (Hz)
−30
−20
−10
0
10
20
30
4.0
3.5
3.0
2.5
2.0
1.5
1.0
0.5
F2 (ppm)

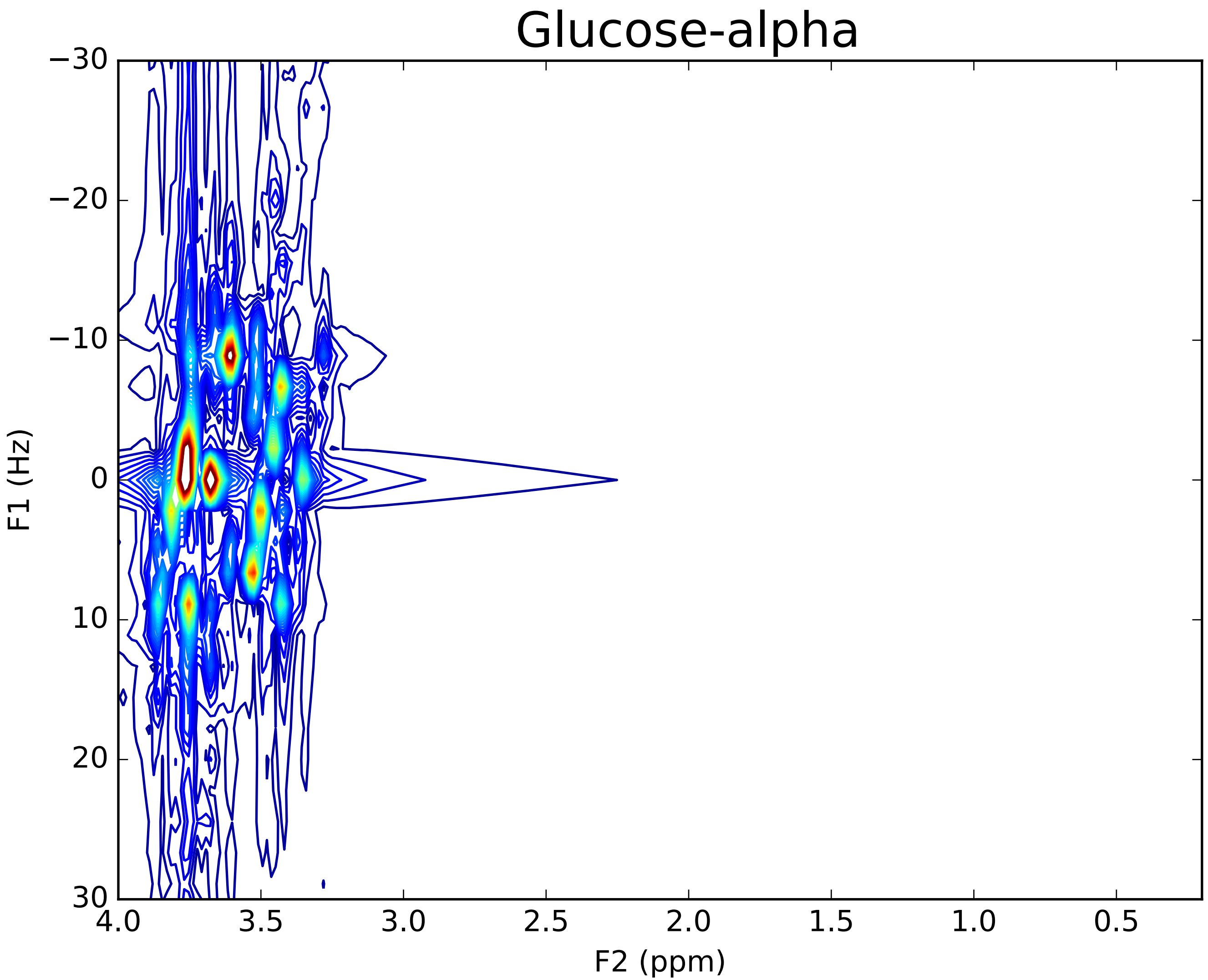

Glucose-alpha
F1 (Hz)
F2 (ppm)
−30
−20
−10
0
10
20
30
4.0
3.5
3.0
2.5
2.0
1.5
1.0
0.5

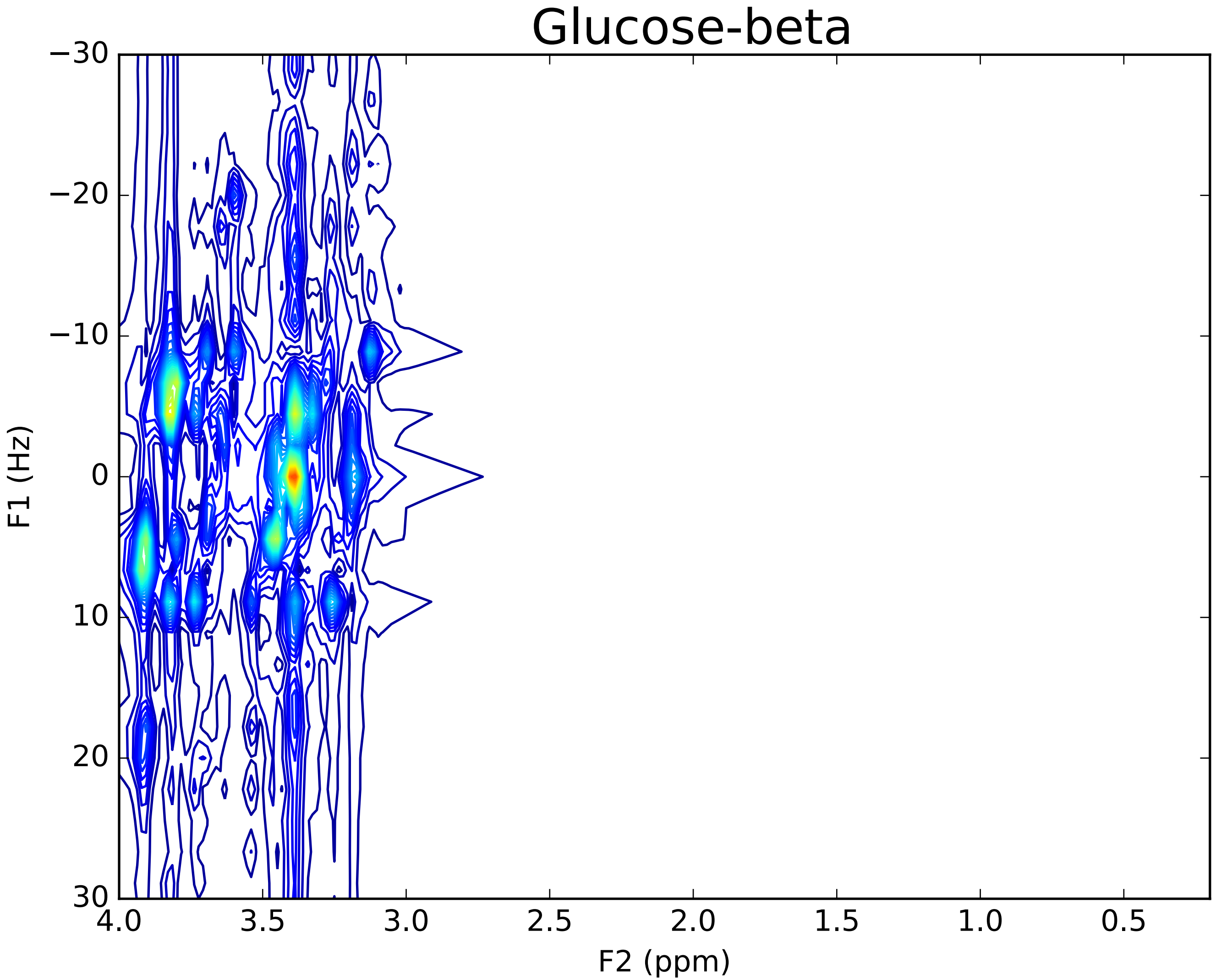

Glucose-beta
−30
−20
−10
0
10
20
30
F1 (Hz)
4.0
3.5
3.0
2.5
2.0
1.5
1.0
0.5
F2 (ppm)

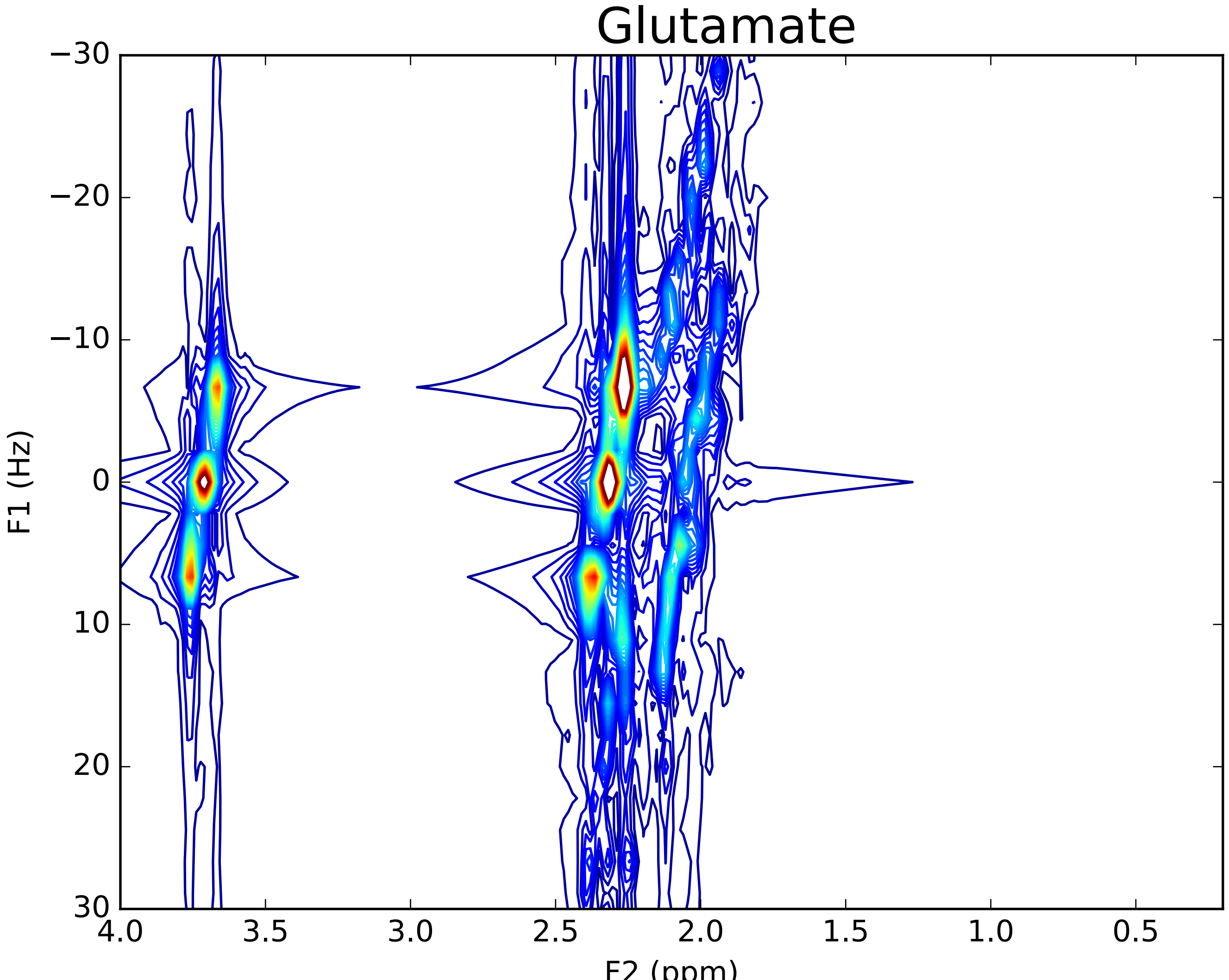

Glutamate
F1 (Hz)
F2 (ppm)
−30
−20
−10
0
10
20
30
4.0
3.5
3.0
2.5
2.0
1.5
1.0
0.5

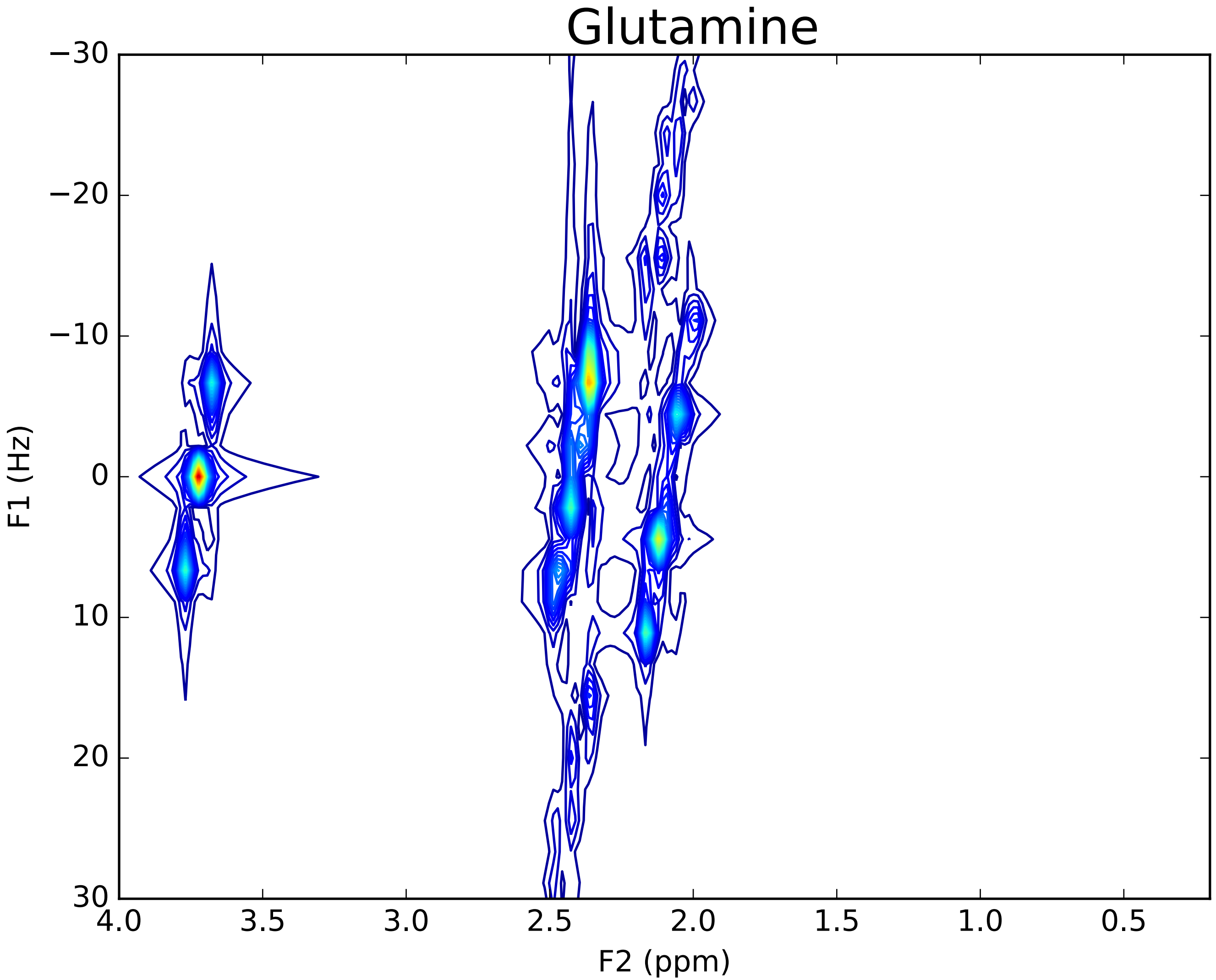

Glutamine
F1 (Hz)
F2 (ppm)
−30
−20
−10
0
10
20
30
4.0
3.5
3.0
2.5
2.0
1.5
1.0
0.5

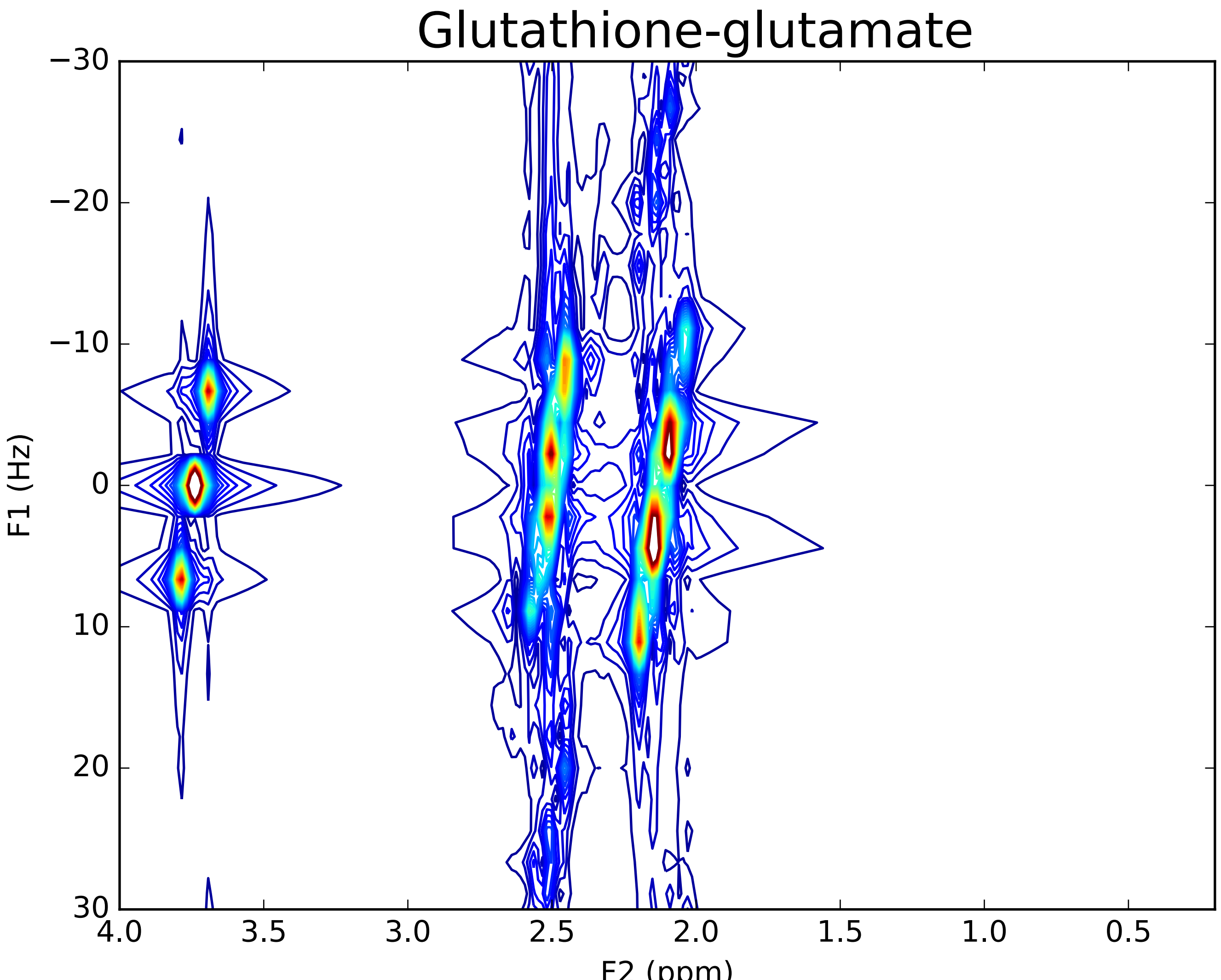

Glutathione-glutamate
F1 (Hz)
F2 (ppm)
−30
−20
−10
0
10
20
30
4.0
3.5
3.0
2.5
2.0
1.5
1.0
0.5

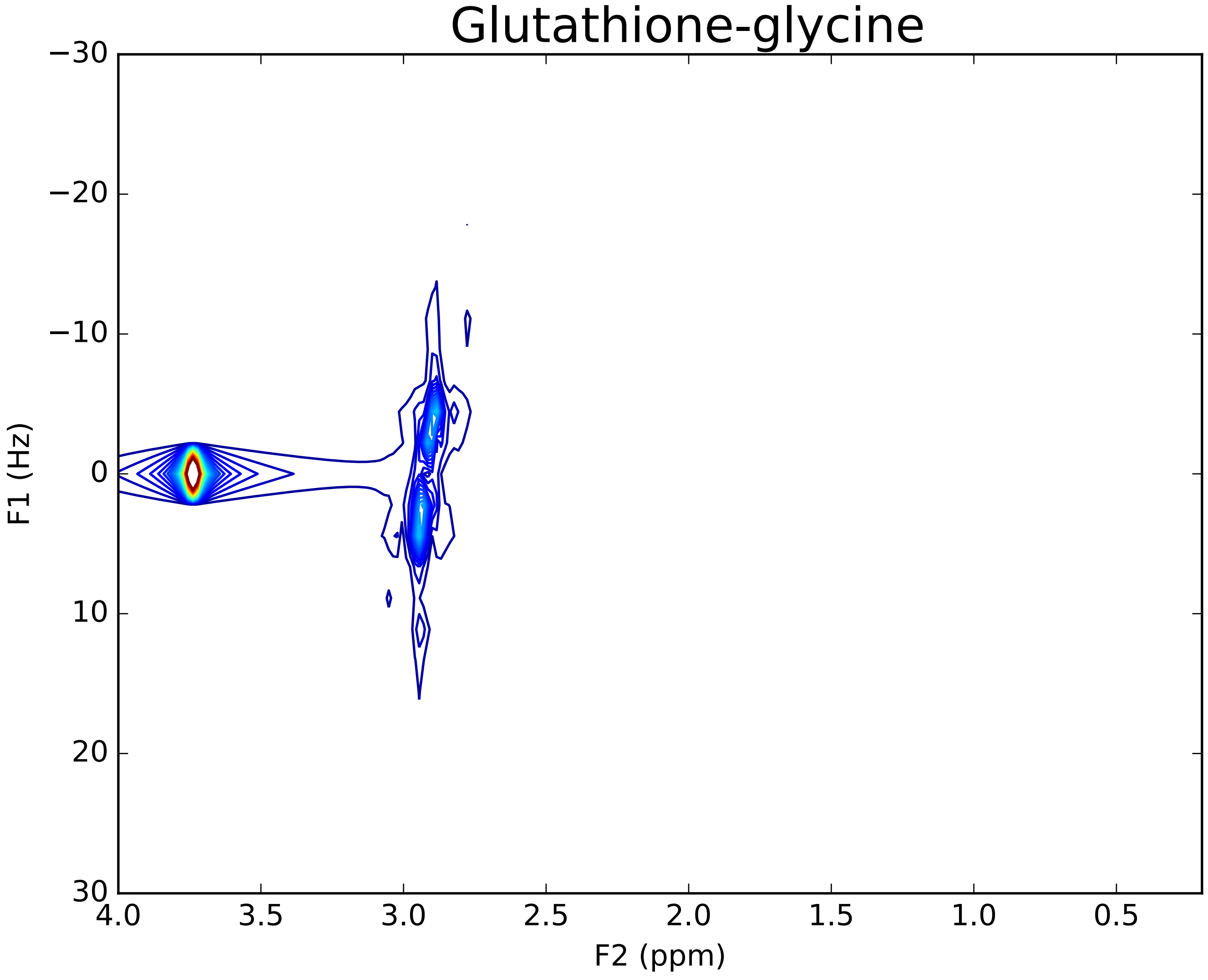

Glutathione-glycine
−30
−20
−10
0
10
20
30
F1 (Hz)
4.0
3.5
3.0
2.5
2.0
1.5
1.0
0.5
F2 (ppm)

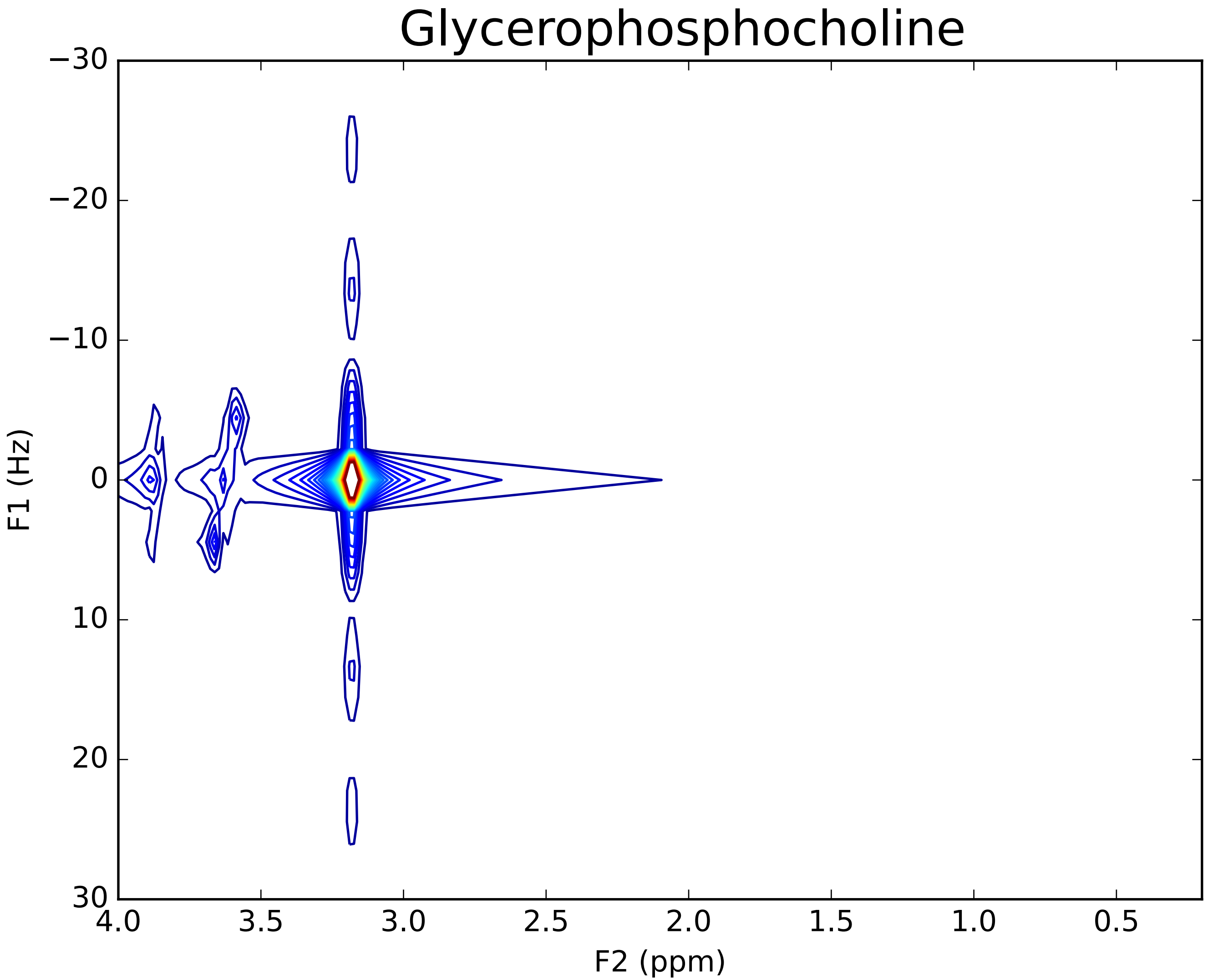

Glycerophosphocholine
F1 (Hz)
F2 (ppm)
−30
−20
−10
0
10
20
30
4.0
3.5
3.0
2.5
2.0
1.5
1.0
0.5

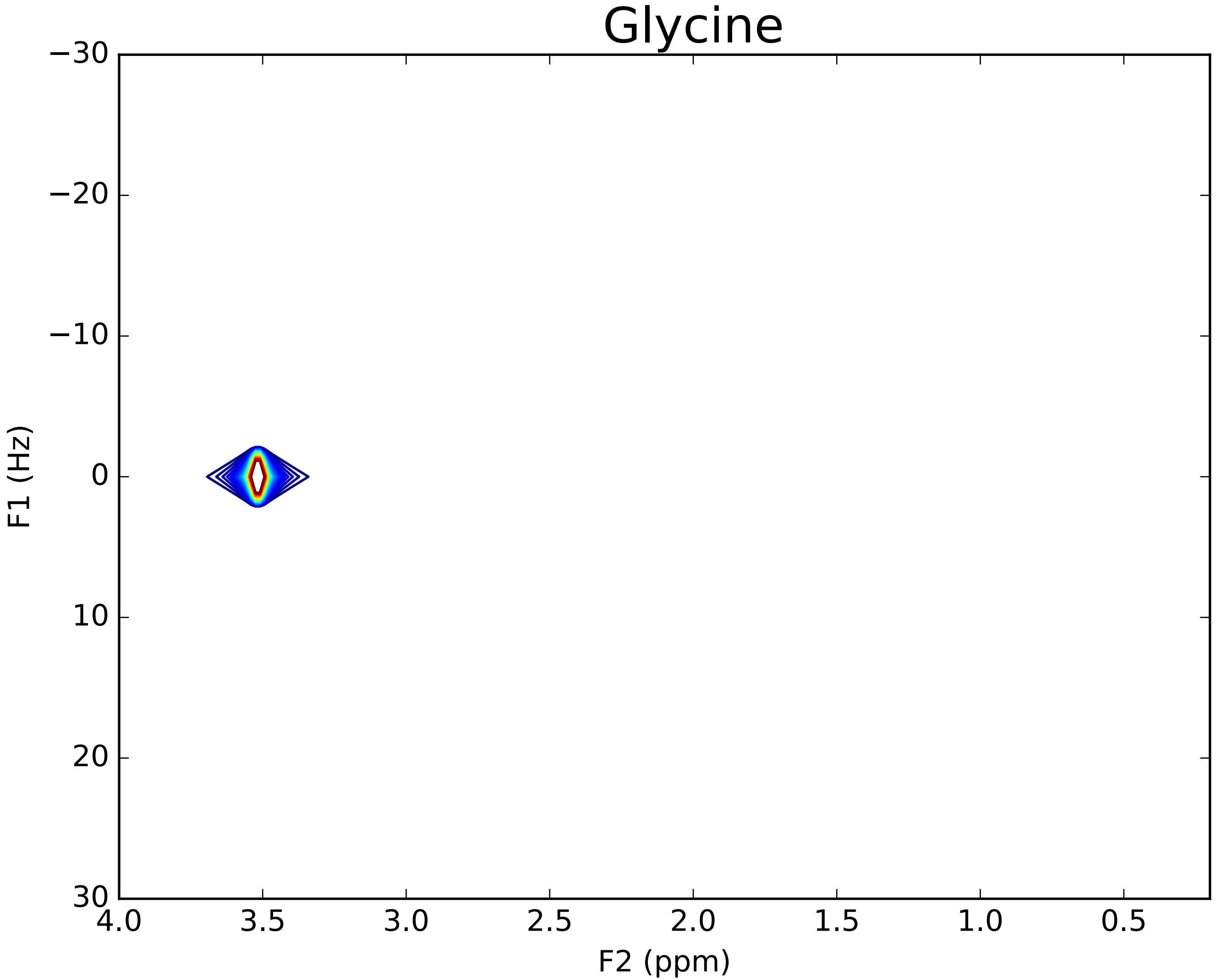

Glycine
F1 (Hz)
F2 (ppm)
−30
−20
−10
0
10
20
30
4.0
3.5
3.0
2.5
2.0
1.5
1.0
0.5

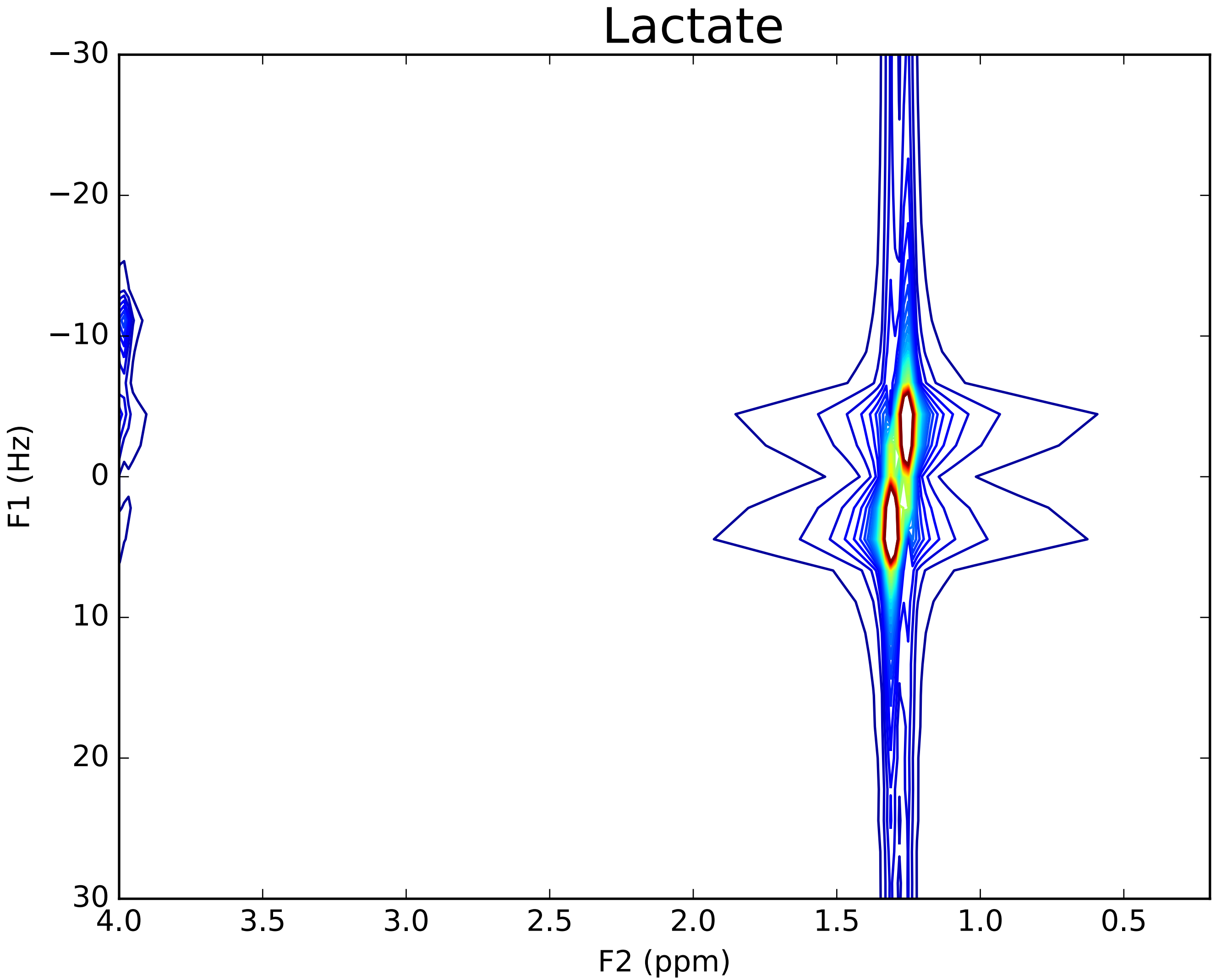

Lactate
−30
−20
−10
0
10
20
30
F1 (Hz)
4.0
3.5
3.0
2.5
2.0
1.5
1.0
0.5
F2 (ppm)

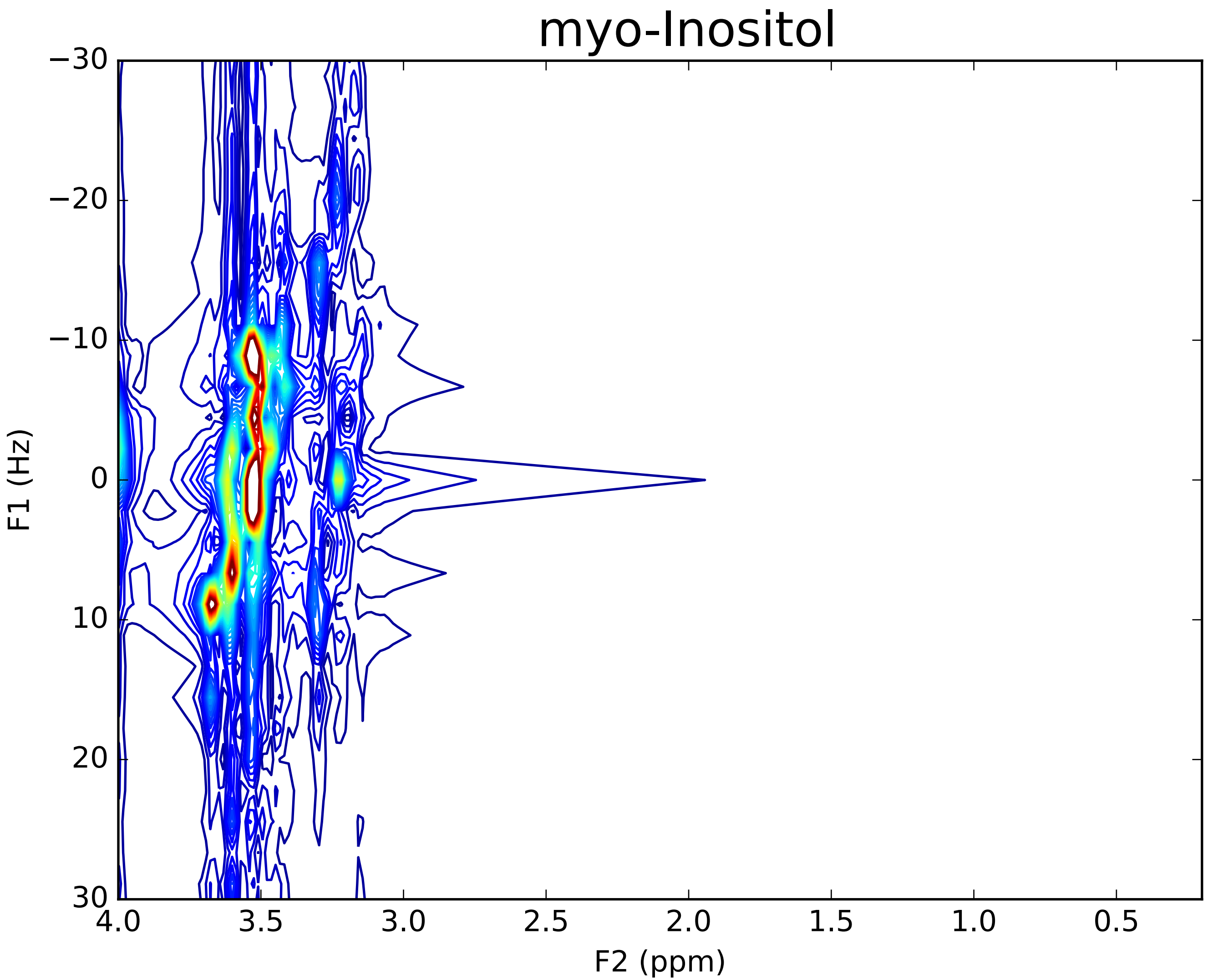

myo-Inositol
−30
−20
−10
0
10
20
30
F1 (Hz)
4.0
3.5
3.0
2.5
2.0
1.5
1.0
0.5
F2 (ppm)

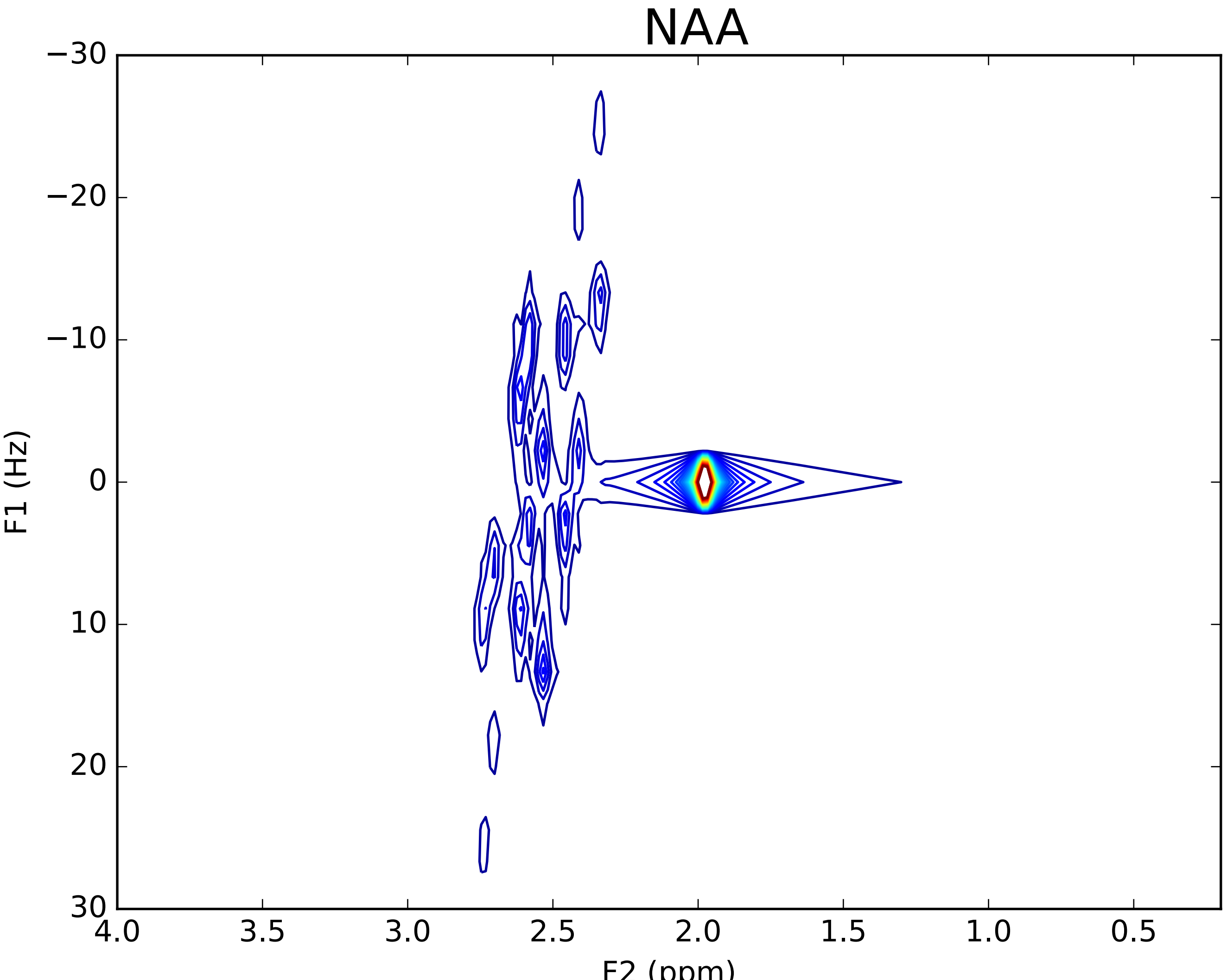

NAA
−30
−20
−10
0
10
20
30
F1 (Hz)
4.0
3.5
3.0
2.5
2.0
1.5
1.0
0.5
F2 (ppm)

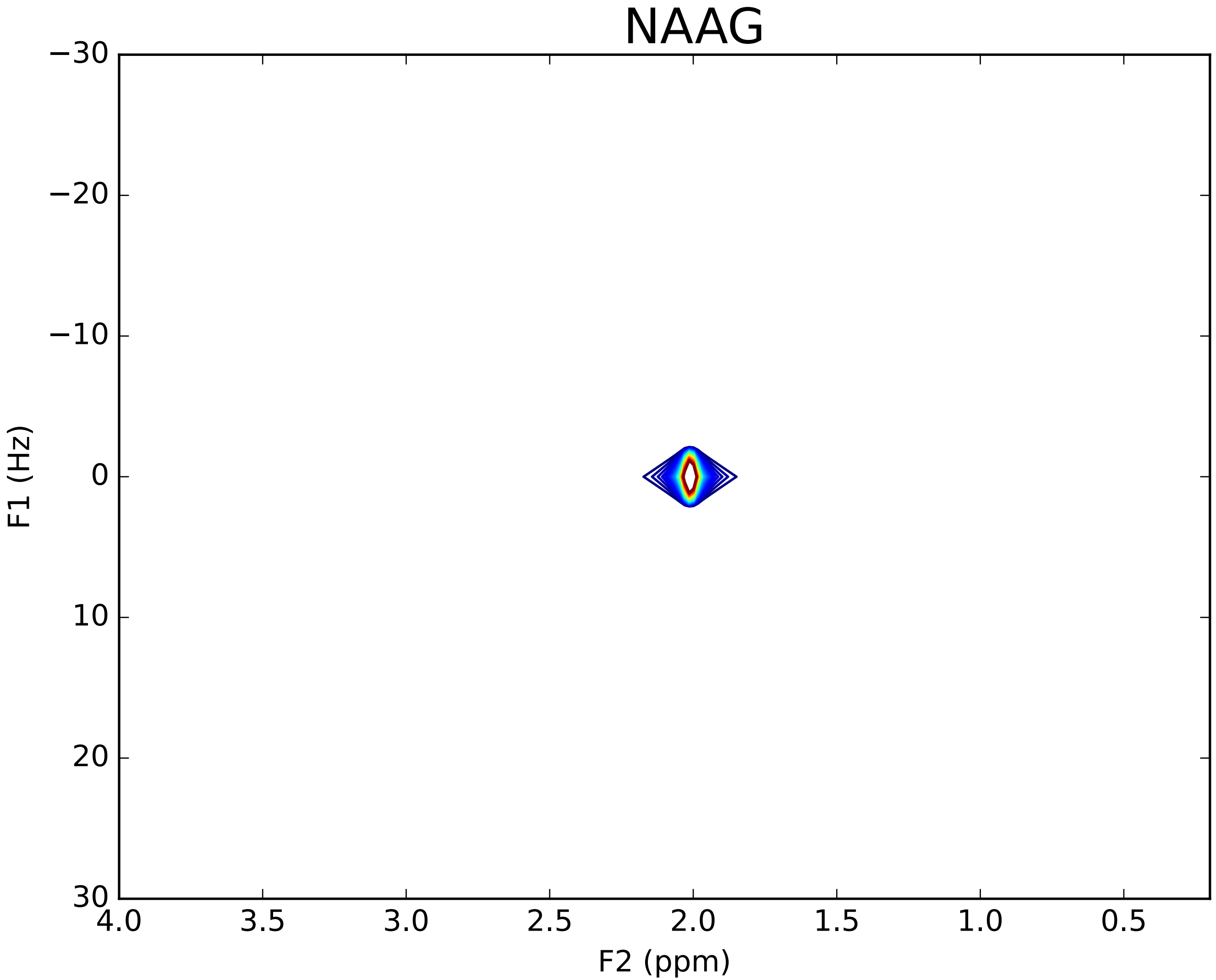
NAAG
F1 (Hz)
F2 (ppm)
−30
−20
−10
0
10
20
30
4.0
3.5
3.0
2.5
2.0
1.5
1.0
0.5

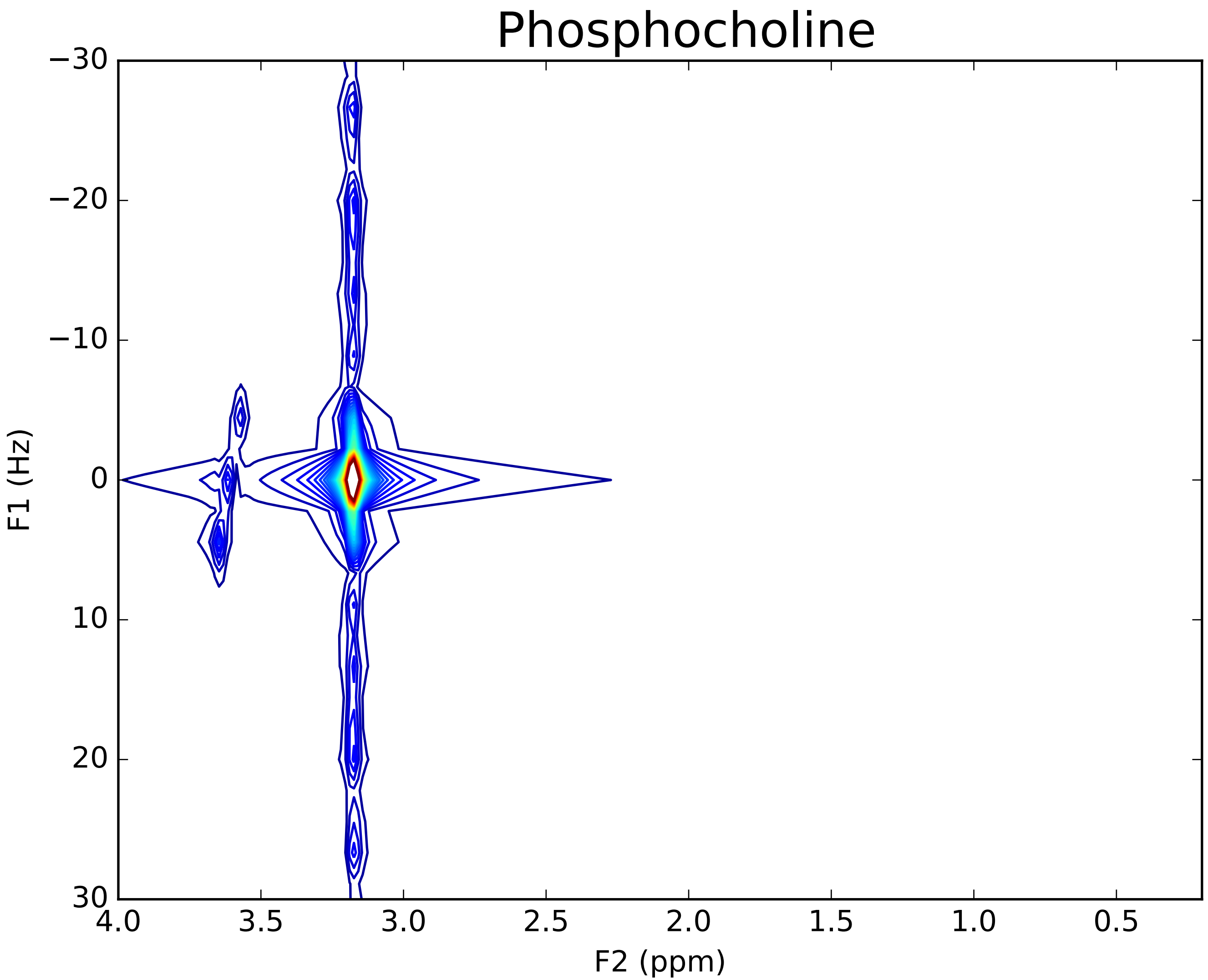
Phosphocholine
F1 (Hz)
F2 (ppm)
−30
−20
−10
0
10
20
30
4.0
3.5
3.0
2.5
2.0
1.5
1.0
0.5

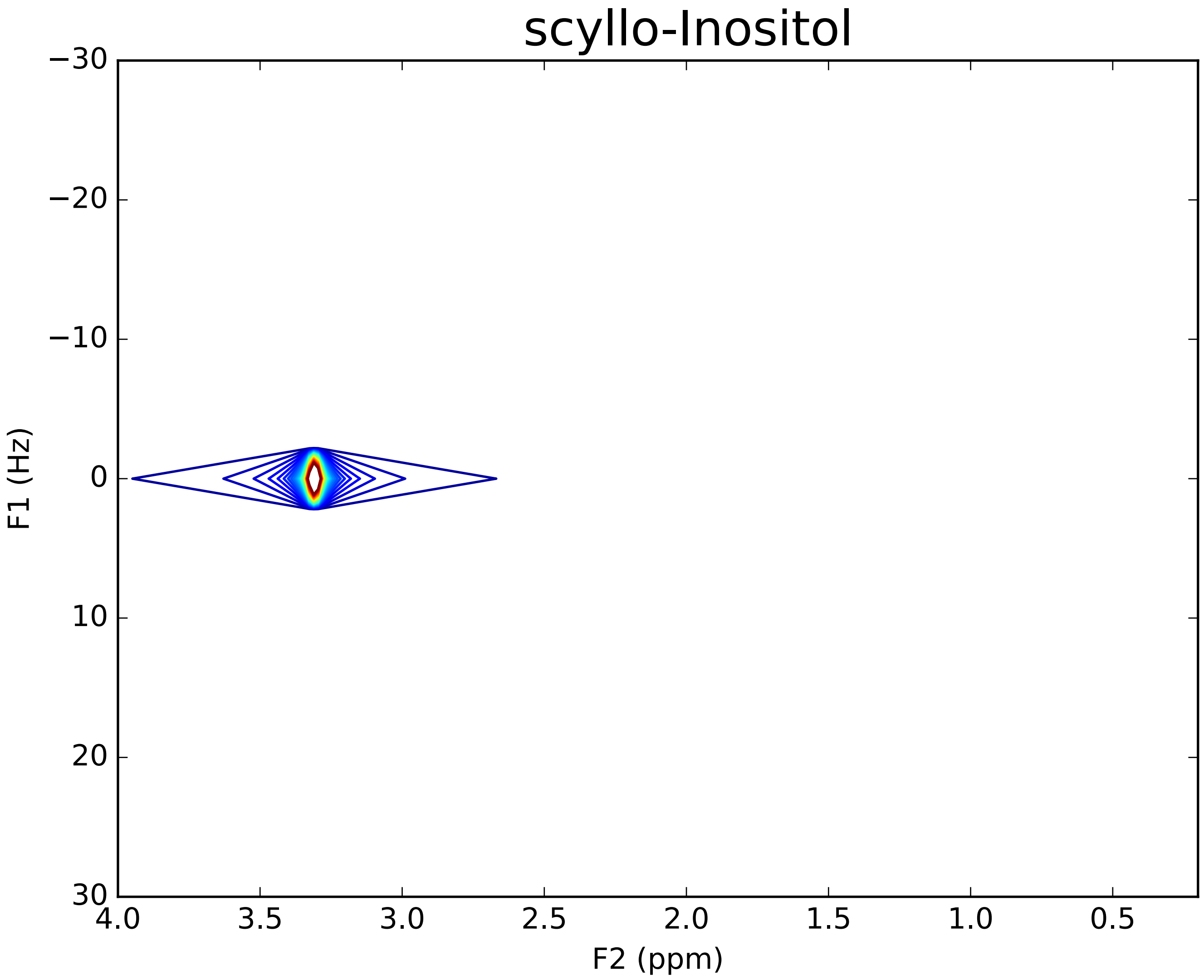
scyllo-Inositol
F1 (Hz)
F2 (ppm)
−30
−20
−10
0
10
20
30
4.0
3.5
3.0
2.5
2.0
1.5
1.0
0.5

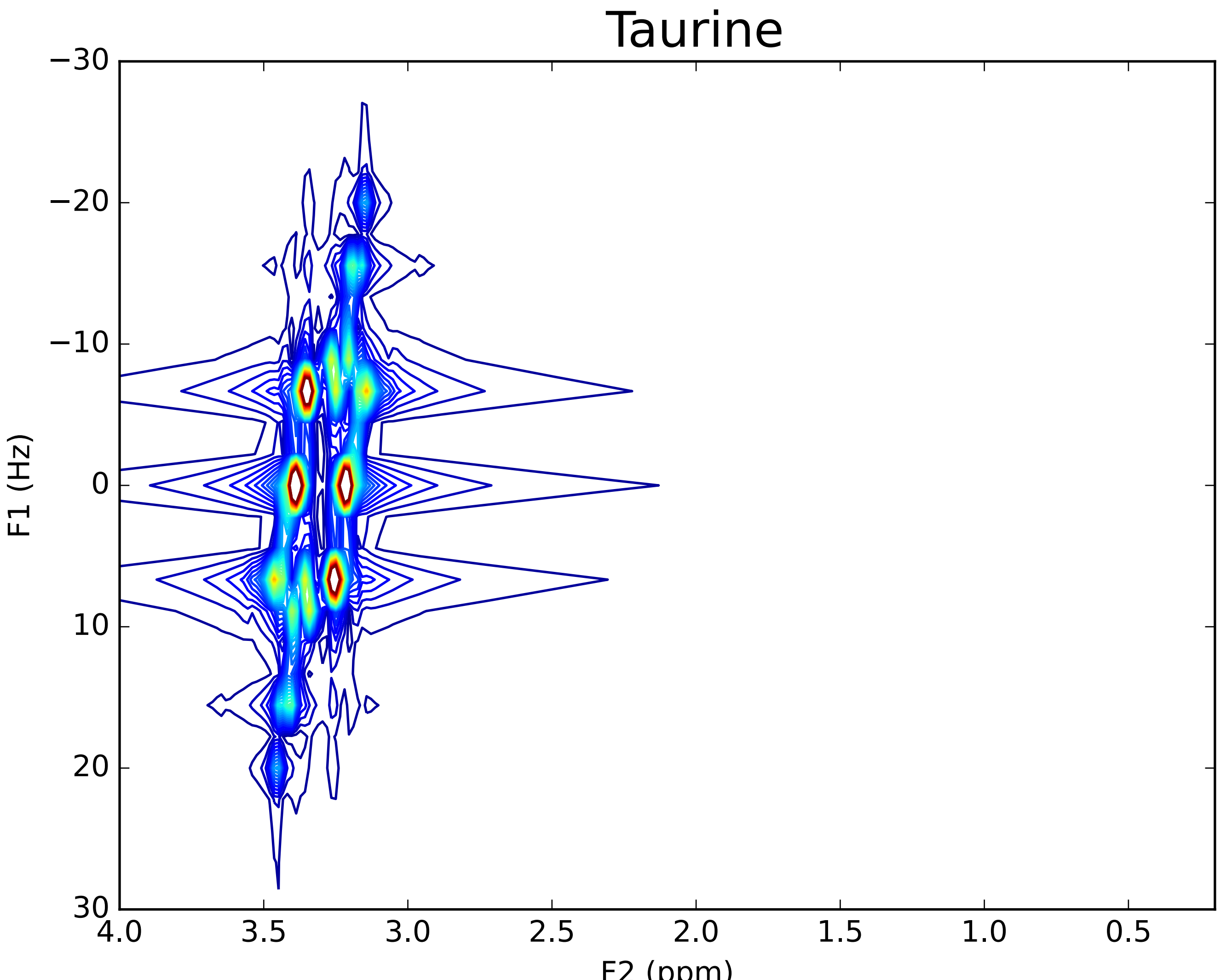

Taurine
−30
−20
−10
0
10
20
30
F1 (Hz)
4.0
3.5
3.0
2.5
2.0
1.5
1.0
0.5
F2 (ppm)
